# Advancing cancer therapy: Nanomaterial-based encapsulation strategies for enhanced delivery and efficacy of curcumin

**DOI:** 10.1016/j.mtbio.2025.101963

**Published:** 2025-06-09

**Authors:** Yuxing Yan, Kulsoom, Yanbo Sun, Yingjie Li, Zhenlong Wang, Li Xue, Fu Wang

**Affiliations:** a Department of Urology, The Second Affiliated Hospital of Xi'an Jiaotong University, Xi'an, Shaanxi, 710004, China; b Institute of Medical Engineering, School of Basic Medical Sciences, Xi'an Jiaotong University, Xi'an, 710061, China

**Keywords:** Curcumin, Nanocapsules, Cancer therapy, Drug delivery

## Abstract

Curcumin, a polyphenolic compound derived from *Curcuma longa*, has gained significant attention as a potential anticancer agent due to its anti-inflammatory, antioxidant, and antitumor properties. Despite its therapeutic potential, the clinical application of curcumin is limited by its poor aqueous solubility, rapid metabolism, and limited bioavailability. To address these limitations, various nanomaterial-based encapsulation strategies have been developed, including polymeric nanoparticles, liposomes, solid lipid nanoparticles, micelles, dendrimers, and hybrid nanomaterials. These formulations aim to improve curcumin's solubility, stability, cellular uptake, and controlled release, thereby enhancing its targeted delivery to tumor sites. Such approaches not only reduce systemic toxicity but also improve therapeutic efficacy. Recent studies demonstrate that curcumin-loaded nanocarriers exhibit enhanced antitumor effects, selective cytotoxicity toward cancer cells, and minimized side effects. However, challenges such as achieving tissue specificity, evaluating potential toxicity, and the need for thorough clinical validation persist. Future research should prioritize the development of tissue-specific delivery systems, assess safety profiles, and ensure biocompatibility to optimize curcumin's clinical efficacy. This review provides an overview of the latest advancements in curcumin nanocapsules, critically comparing their advantages and limitations in cancer therapy.

## Graphical abstract

**Figure:**
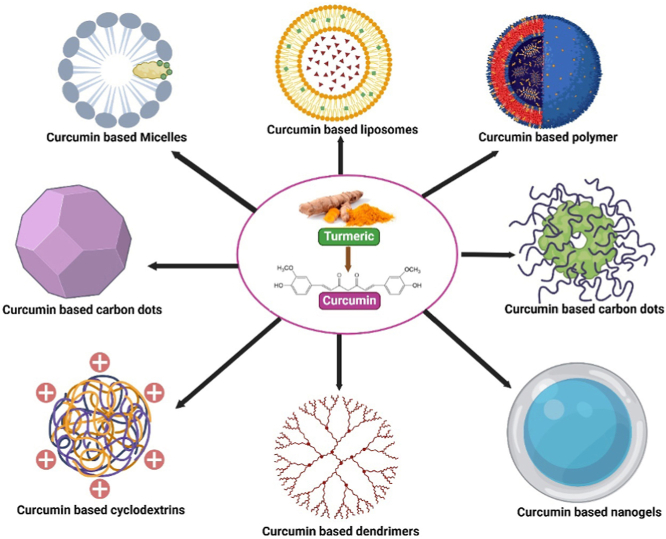

## 1 Introduction

Cancer remains the second leading cause of death globally [1], with its incidence steadily rising due to complex genetic mutations and epigenetic alterations [2], influenced by environmental factors such as chronic inflammation, radiation, and chemical exposure [3]. Despite advances in oncology, the management of malignant tumors continues to face significant challenges, particularly in addressing drug resistance, systemic toxicity, and tumor heterogeneity [4,5]. Current therapeutic modalities—surgery, radiation, chemotherapy, targeted therapy, and immunotherapy—have improved survival outcomes, yet metastatic and recurrent cancers often remain refractory to treatment [6].

In recent years, complementary and alternative therapies, including Traditional Chinese Medicine (TCM), have garnered attention, especially in Asia, for their potential to modulate immune responses and improve quality of life in cancer patients [7,8]. Among bioactive compounds derived from natural products, curcumin, a polyphenolic constituent of *Curcuma longa*, has been extensively investigated for its diverse pharmacological properties, encompassing antioxidant, anti-inflammatory, antimicrobial, and anticancer effects [9].

Curcumin exhibits pleiotropic activity by modulating multiple molecular targets involved in tumorigenesis, including transcription factors (NF-κB, STAT3), growth factors, inflammatory mediators (TNF-α, IL-6), apoptotic regulators (Bcl-2 family), and key signaling pathways such as PI3K/Akt, MAPK, and Wnt/β-catenin [8,10]. These multifaceted interactions confer curcumin with potential for inhibiting tumor initiation, progression, invasion, angiogenesis, and metastasis.

However, despite extensive preclinical evidence supporting its anticancer potential, the clinical translation of curcumin remains limited, primarily due to its poor aqueous solubility, rapid metabolism, low systemic bioavailability, and instability under physiological conditions [11]. Pharmacokinetic studies have demonstrated that even high oral doses of curcumin yield subtherapeutic plasma concentrations, undermining its clinical efficacy [[12], [13], [14]]. Moreover, curcumin's chemical instability under neutral and alkaline conditions further restricts its therapeutic applicability.

**Fig. 1:**
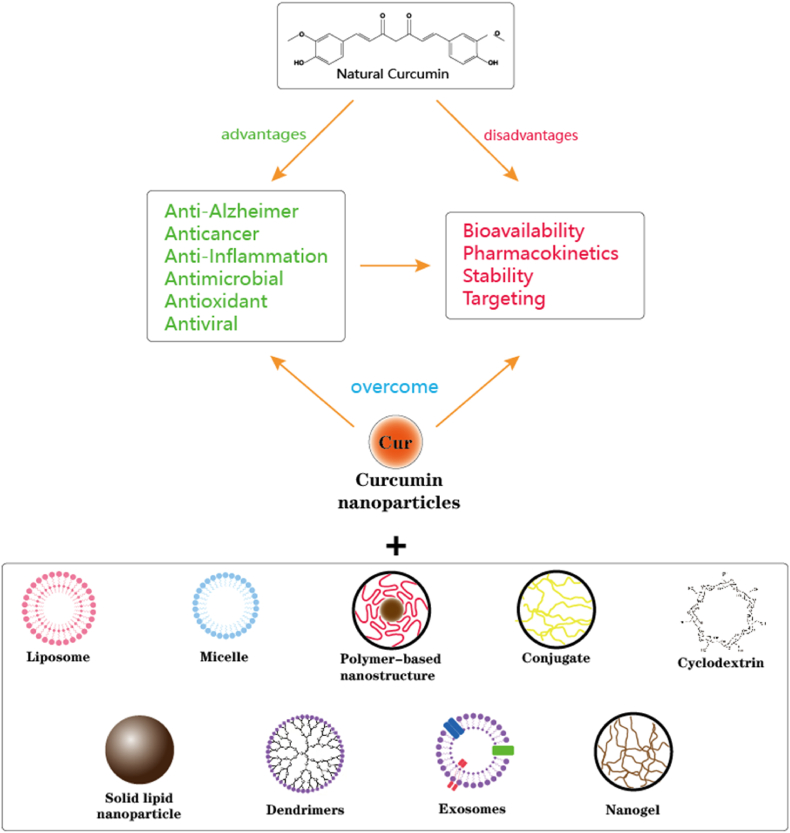
Nano-based curcumin formulations substantially impact pharmaceutical applications, effectively treating various human disorders due to their anti-cancer, antioxidant, antimicrobial, anti-inflammatory, and anti-Alzheimer properties. These nanoformulations can overcome curcumin's weak hydrophobicity, poor stability, and low cellular bioavailability.

To overcome these challenges, considerable research efforts have been directed toward improving curcumin's bioavailability and targeted delivery. Recent strategies include the development of nanocarrier-based delivery systems, such as liposomes, polymeric nanoparticles, dendrimers, micelles, and protein-based carriers [15,16] (Fig. 1). These systems enhance curcumin's physicochemical stability, solubility, and controlled release properties while enabling tumor-specific accumulation through passive (enhanced permeability and retention effect (EPR)) and active targeting mechanisms [[17], [18], [19]].

Given these advancements, this review aims to comprehensively explore curcumin's modulation of key oncogenic signaling pathways, highlight the pharmacokinetic barriers hindering its clinical application, and critically assess nanomaterial-based encapsulation strategies designed to enhance its therapeutic efficacy in cancer treatment. Although this review primarily emphasizes curcumin-based nano-encapsulation strategies, comparative insights from other phytochemical nanomedicines are also discussed to broaden the translational relevance for the wider drug delivery and nanomedicine community.

### 1.1 Physical properties of curcumin

Curcumin, a yellow polyphenolic compound derived from the rhizomes of *Curcuma longa*, has long been recognized for its health benefits in herbal medicine [6]. It was first isolated by Vogel and Pelletier, who identified a substance with "yellow coloring properties" from turmeric, naming it curcumin [15]. Chemically, curcumin belongs to the diarylheptanoid class and predominantly exists in three curcuminoid forms: CUR I (curcumin), CUR II (demethoxycurcumin), and CUR III (bisdemethoxycurcumin). The chemical structures of these curcuminoids differ primarily in their methoxy group substitutions, which directly influence their solubility, stability, and biological activities. The three primary curcuminoids are: CUR I (1,7-bis-4-hydroxy-3-methoxyphenyl-hepta-1,6-diene-3,5-dione, approximately 77 %), CUR II (dimethoxy CUR, 1,4-hydroxy-3-methoxyphenyl-7,4-hydroxyphenyl-hepta-1,6-diene-3,5-dione, about 17 %), and CUR III (bisdemethoxy CUR, 1,7-bis-4-hydroxyphenyl-hepta-1,6-diene-3,5-dione, around 3 %) [16]. CUR I, which falls under the Biopharmaceutical Classification System (BCS) Class IV [17], has the molecular formula C21H20O6, a molecular weight of 368.37 g/mol, and a melting point of 183 °C. [18]. Table 1 showed the comparison of key physicochemical properties of curcumin derivatives (CUR I, CUR II, CUR III). Curcumin shows limited solubility in water at neutral and acidic pH but is soluble in organic solvents such as methanol, ethanol, acetone, and dimethyl sulfoxide due to its lipophilic nature, with a log P value of approximately 3.0 [[19], [20], [21]].

**Table 1:** Comparison of key physicochemical properties of curcumin derivatives (CUR I, CUR II, CUR III).

Property	CUR I (Curcumin)	CUR II (Demethoxycurcumin)	CUR III (Bisdemethoxycurcumin)
Molecular Formula	C_21_H_20_O_6_	C_20_H_18_O_5_	C_19_H_16_O_4_
Molecular Weight (g/mol)	368.38	338.35	308.32
Aqueous Solubility (μg/mL)	11	8	5
Log P (Partition Coefficient)	3.2	3.0	2.8
Chemical Stability (Half-life in PBS, pH 7.4, 37 °C)	1–2 h	2.5 h	3–4 h
Melting Point (°C)	183–186	175–178	178–181
Polarity (Relative)	Low	Moderate	Higher

Curcumin is chemically unstable in aqueous and neutral pH environments, undergoing rapid hydrolytic degradation to ferulic acid, vanillin, and other phenolic byproducts [18]. Additionally, it is highly susceptible to oxidative degradation and photodegradation under ambient light, limiting its shelf life and therapeutic application. In physiological pH (7.4) at 37 °C, curcumin exhibits a half-life of approximately 1–2 h, while its analogs demonstrate marginally better stability. Curcumin is practically insoluble in water (11 μg/mL) but exhibits higher solubility in organic solvents such as methanol, ethanol, acetone, and dimethyl sulfoxide. Its lipophilicity, denoted by a log P value of 3.2, underpins its affinity for lipid bilayers and hydrophobic nanocarrier systems, but concurrently contributes to its poor oral bioavailability [[19], [20], [21]].

Curcumin exists in keto-enol tautomeric forms, with the enol form predominating in non-polar solvents, while the diketone form is favored in polar and acidic media [22]. This tautomeric behavior influences curcumin's binding interactions, absorption, and reactivity in biological systems. Accurate detection and quantification of curcumin in biological samples and nanoformulations typically require high-performance liquid chromatography (HPLC), liquid chromatography–mass spectrometry (LC–MS), and nuclear magnetic resonance (NMR) spectroscopy [23,24]. These methods ensure precise monitoring of curcumin's pharmacokinetics, stability, and degradation products during preclinical and clinical evaluations. The poor aqueous solubility and instability of curcumin make it an ideal candidate for nanoencapsulation strategies [22]. Its hydrophobicity allows efficient incorporation into lipophilic cores of nanocarriers such as liposomes, polymeric nanoparticles, micelles, and dendrimers, which enhances its aqueous dispersibility, stability, and therapeutic efficacy.

### 1.2 Biological properties of curcumin

**Fig. 2:**
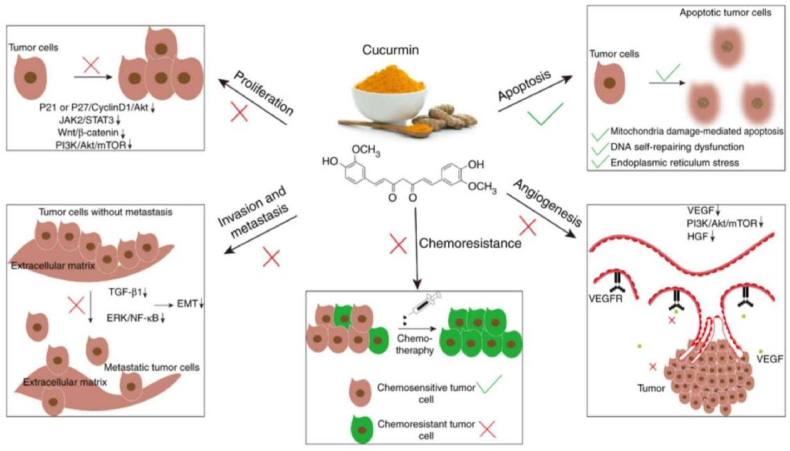
Curcumin inhibits tumor growth by suppressing proliferation, angiogenesis, invasion, and metastasis, and increase drug sensitivity and apoptosis [29].

Curcumin exhibits diverse biological activities such as anti-inflammatory, antioxidant, anticoagulant, anti-hyperlipidaemic, anti-atherosclerotic, anti-mutagenic, and anti-tumor activities [23]. Curcumin's multifaceted therapeutic potential across various diseases, recent research has particularly emphasized investigating its anticancer properties and elucidating the underlying mechanisms [24,25], the inhibitory effects of curcumin on a diverse range of cancers, including breast, lung, colon, and liver cancers. This naturally occurring compound exhibits potent anti-cancer properties by modulating cellular signal transduction pathways and influencing key processes such as proliferation, apoptosis, and migration in cancer cells [26] (Fig. 2).

Curcumin demonstrates significant antitumor activity; however, its inherent dissolution rate, high metabolic activity, low oral bioavailability, and poor pharmacokinetic properties restrict its potential as a highly effective tumor therapy. Recent studies have indicated that nanomaterials' EPR effects offer novel approaches to improve the accumulation of chemotherapeutic drugs at the tumor site. By utilizing nanocarriers such as liposomes, carbon nanotubes, dendritic polymers, and micelles, drug concentrations like cisplatin in tumor tissues have been augmented and widely employed for delivering drugs like SN38, doxorubicin, and paclitaxel while reducing adverse effects [27]. Moreover, nanomaterials offer the advantage of enhancing the solubility, biocompatibility, and drug absorption of chemotherapeutic agents. Nanoencapsulation strategies significantly enhance the pharmacological effects of curcumin. Therefore, curcumin nano-formulations hold immense potential to provide robust support for achieving therapeutic benefits [28].

#### 1.2.1 Regulation of NF-κB pathway by curcumin

The NF-κB family of transcription factors plays a critical role in controlling genes involved in inflammation, proliferation, and survival, with aberrant activation implicated in various cancers [30,31]. Studies show that curcumin has therapeutic potential in oncology by modulating key cell signaling pathways, including EGFR and ERBB2 [[32], [33], [34], [35], [36]]. Curcumin, particularly its analog EF24, is a potent disruptor of this pathway and shows significant anticancer potential [37]. Curcumin inhibits NF-κB activity, leading to cellular apoptotic responses in cancer cells [38]. This inhibition of NF-κB-related pathways often reduces cancer cell proliferation and increases sensitivity to chemotherapy [39]. Curcumin is a potent inhibitor of NF-κB; its synthetic analog EF24 demonstrates even greater efficacy. EF24 exhibits enhanced bioavailability and more potent bioactivity than curcumin, including more substantial anti-cancer properties [40]. EF24 inhibits tumor growth by inducing cell cycle arrest and apoptosis via NF-κB pathway inhibition [37,40]. However, another analog, EF31, has shown even more potent inhibition of NF-κB activity than EF24 and curcumin [41]. Curcumin effectively impedes phosphorylation events within the NF-κB signal transduction pathway, attenuating its expression. Curcumin inhibits TNF-α-induced NF-κB nuclear translocation and DNA binding by blocking IκBα phosphorylation and degradation [42]. Subsequent investigations have revealed that curcumin can downregulate NF-κB-inducible kinase (NIK) and I-κB kinase (IKK), thereby impeding the degradation of I-κB. In experimental studies, curcumin demonstrated anti-proliferative, pro-apoptotic, and anti-metastatic properties in multiple myeloma and mouse melanoma cells by inhibiting IKK activity, thus substantiating its scientific validity [43,44]. Curcumin inhibits the expression of cell cycle protein D1, COX-2, MMP-9, VEGF, and CXCR4, likely by suppressing NF-κB activation. It may hinder cell proliferation by blocking the G1 to S phase transition, reduce inflammation and matrix degradation by inhibiting COX-2 and MMP-9, and suppress angiogenesis by downregulating VEGF and CXCR4. These effects contribute to curcumin's potential in preventing diseases linked to angiogenesis, such as ovarian cancer, by modulating the NF-κB pathway [45].

**Fig. 3:**
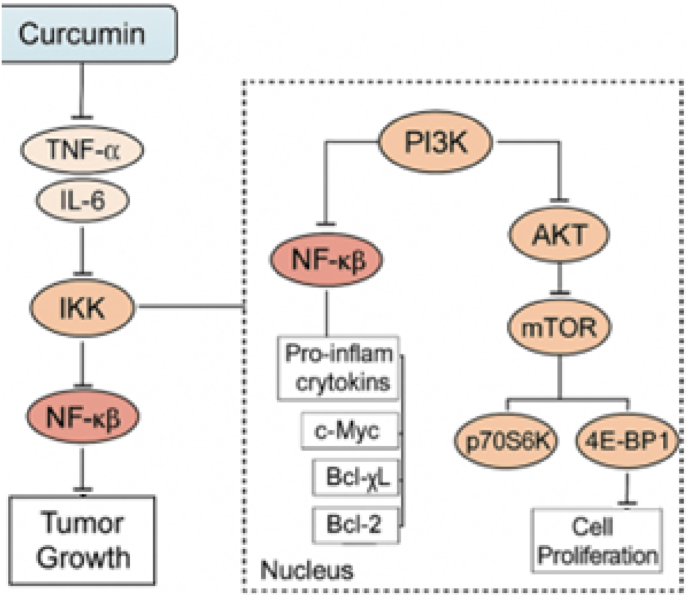
Curcumin modulates NF-κB and PI3K/AKT/mTOR signaling pathways to suppress tumor progression.

Curcumin inhibits the activation of NF-κB by blocking IKK-mediated phosphorylation and degradation of IκBα, thereby preventing nuclear translocation and downregulating pro-inflammatory cytokines (e.g., TNF-α, IL-6) and survival genes (e.g., Bcl-2, cyclin D1). Concurrently, curcumin suppresses PI3K/AKT/mTOR signaling by reducing PI3K activity, AKT phosphorylation, and mTORC1-dependent phosphorylation of downstream effectors (p70S6K, 4E-BP1), leading to inhibition of cell proliferation and induction of apoptosis (Fig. 3).

#### 1.2.2 Regulation of PI3K/AKT/mTOR pathway by curcumin

**Fig. 4:**
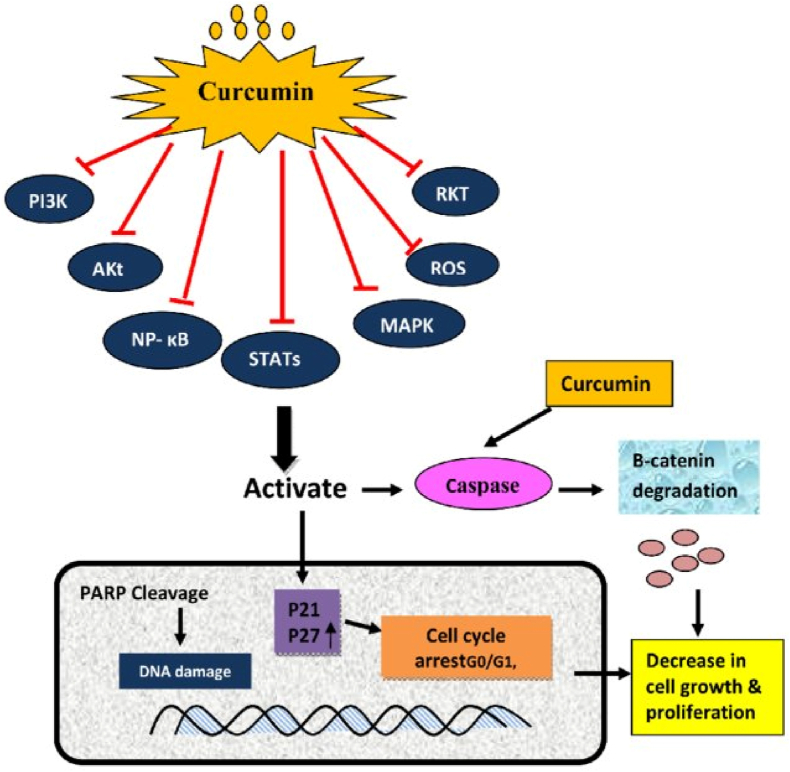
Curcumin-based nanoparticles regulate the PI3K/Akt, MAPK, Wnt/β-catenin, JAK/STAT, p53, and NF-κB pathways, while inhibiting oncogenic miRNAs and increasing proto-oncogenic non-coding RNAs, contributing to anticancer effects. These nanoparticles inhibit cancer cell proliferation, EMT, invasion, metastasis, and drug resistance, while promoting apoptosis [49].

The PI3K/Akt/mTOR pathway is frequently hyperactivated in malignancies, promoting tumor growth and survival [46,47]. In malignant tumors, the balance between upstream activators and downstream effectors of mTOR is disrupted, further confirming the critical role of mTOR in cancer development [48]. Curcumin-based nanoparticles have shown promising anticancer effects by regulating the PI3K/AKT/mTOR pathway (Fig. 4) [49]. Studies have demonstrated that curcumin inhibits this signaling pathway, leading to reduced cell proliferation and increased apoptosis in various cancer types [50]. Solid lipid curcumin particles (SLCP) demonstrate stronger apoptotic and anti-carcinogenic effects than natural curcumin in glioblastoma multiforme (GBM) cells. SLCP treatment increases autophagy markers, decreases mitophagy markers, and inhibits the PI3K-Akt/mTOR pathway, resulting in downregulation of cell survival markers and upregulation of cell death markers [51]. In non-small cell lung cancer (NSCLC) cells, curcumin inhibited cell viability in a time- and dose-dependent manner while inducing apoptosis and autophagy. The mechanism involved substantial downregulation of the PI3K/Akt/mTOR pathway. Combining curcumin with mTOR inhibitor rapamycin or PI3K/Akt inhibitor LY294002 augmented these effects, suggesting a synergistic anticancer action [52]. Curcumin exerts notable anti-apoptotic effects in various malignant tumors by modulating the PI3K/Akt/mTOR signaling pathway [46]. Curcumin effectively inhibits proliferation and induces apoptosis in human non-small cell lung cancer (NSCLC) cells by suppressing the PI3K/Akt pathway and upregulating miR-192-5p [53]. On the other hand, curcumin can also inhibit neutrophil elastase-induced lung tumor proliferation by modulating the PI3K/Akt pathway and enhancing the expression of α1-antitrypsin in vitro and in vivo, an enzyme released in response to inflammation and associated with tumor growth and metastasis [54]. Curcumin downregulates Akt protein in a dose- and time-dependent manner in breast cancer cells, inducing autophagy and inhibiting the ubiquitin-proteasome pathway. It also suppresses cell growth by downregulating Akt and MAPK expression. The PI3K/Akt-SKP2-Cip/Kips pathway regulates sensitivity differences between MCF-7 and MDA-MB-231 cells [55]. Overall, curcumin enhances apoptosis and autophagy by blocking the PI3K/Akt pathway [56]. Moreover, combining curcumin with PI3K inhibitors synergistically induces apoptotic effects in MCF-7 cells, offering a novel strategy for breast cancer treatment [[55], [56], [57]]. The PI3K/Akt signaling pathway is often dysregulated in lymphomas, contributing to tumorigenesis and increased resistance to radiotherapy [49]. Qiao et al. demonstrated that curcumin effectively suppresses both persistent and radiation-induced PI3K/Akt expression in human Burkitt lymphoma cells [58].

#### 1.2.3 Regulation of STAT3 pathway by curcumin

The STAT3 (Signal Transducer and Activator of Transcription 3) is constitutively activated in various tumor types and [59], contributes to oncogenesis by promoting survival, proliferation, and immune evasion [60]. Curcumin inhibits STAT3 phosphorylation, DNA binding, and transcriptional activity in multiple cancer models [[61], [62], [63], [64]]. Synthetic analogs such as FLLL11 and FLLL12 exhibit enhanced potency, effectively suppressing STAT3 signaling more efficiently than curcumin [[61], [62], [63], [64]].

Curcumin exerts anticancer effects, in part, by regulating the STAT3 pathway. It inhibits STAT3 phosphorylation, DNA binding, and transcriptional activity, which are essential for cancer cell growth and migration [65]. Synthetic analogs, such as FLLL11 and FLLL12, have been developed to enhance curcumin's potency, showing more effective inhibition of STAT3 signaling than curcumin alone [66]. Cucumber-derived nanovesicles (CDNVs) have also shown promise in cancer therapy by suppressing STAT3 activation [67]. These plant-derived nanoparticles offer a cost-efficient and sustainable alternative to traditional drug isolation methods. Curcumin can be a molecular target for inhibiting STAT3 in diverse tumor types directly or indirectly via suppression of IL-6 signaling [[61], [62], [63], [64]]. Curcumin effectively inhibits STAT3 activation in cell lines, animal models, and clinical studies [68]. It suppresses both constitutive and inducible STAT3 activation in human multiple myeloma cells and, when combined with 5-fluorouracil (5-FU), overcomes chemotherapy resistance in gastric cancer cells. Additionally, curcumin enhances chemotherapy by sensitizing HNSCC cells to cisplatin via STAT3 inhibition [69].

#### 1.2.4 Regulation of miRNAs by curcumin

Curcumin-based nanoparticles have shown promising anticancer effects, including through the regulation of miRNAs [70]. Curcumin exerts its anti-cancer function by modulating microRNA (miRNA) expression [26]. Curcumin inhibits cancer cell proliferation and promotes apoptosis by upregulating oncogenic miRNAs like miR-15a, miR-34a, and miR-181b. This effect is mediated through the regulation of key signaling pathways, including Akt, p53, PTEN, and Bcl-2 [26]. Curcumin selectively regulates miRNAs by binding to their specific sequences, affecting their stability and function. This precise modulation enables curcumin to target cancer cells while sparing normal cells, offering promising anti-cancer effects and new possibilities for treatment [26].

**Fig. 5:**
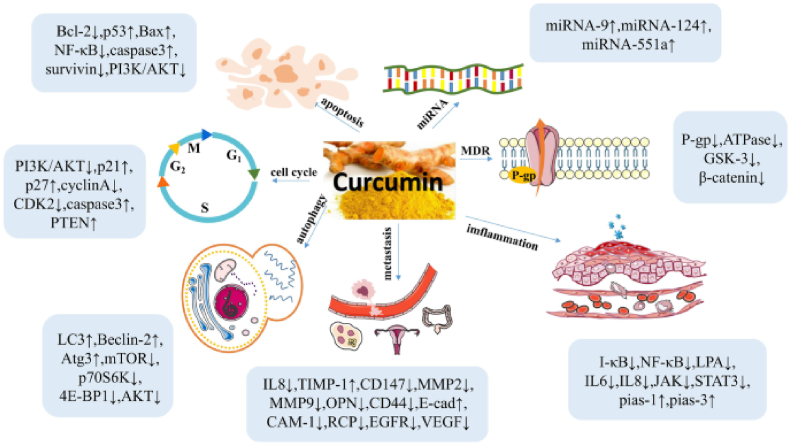
Curcumin inhibits tumor growth by regulating the miRNA level and cell cycle [71].

Cell proliferation drives tumor growth by increasing cell numbers, with abnormal proliferation being crucial for tumor development and spread. Changes in cell cycle-related proteins are key indicators of cell proliferation, and these processes enhance curcumin's anticancer efficacy [26,61,62]. Studies show a strong link between miRNAs and cancer cell proliferation and apoptosis (Fig. 5). The PI3K/Akt pathway is crucial for tumor cell survival, proliferation, and apoptosis [71]. Curcumin inhibits proliferation and induces apoptosis in NSCLC cells by upregulating miR-192-5p, blocking tumor growth in the G2/M phase, and downregulating the PI3K/Akt pathway [53]. The tumor suppressor PTEN regulates this pathway by dephosphorylating PIP3, inhibiting cell proliferation, survival, and migration. Curcumin increases PTEN expression by inhibiting miR-21 [72], a microRNA that regulates the expression of various genes by binding to the 3′UTR region of target mRNAs to inhibit their translation or promote their degradation. Curcumin increased the level of PTEN by inhibiting the expression of miR-21 through the following mechanism of action: curcumin binds to miR-21. It prevents it from binding to the 3′UTR region of PTEN mRNA, thus releasing the inhibitory effect of miR-21 on PTEN [72]. The increase of PTEN expression inhibited the PI3K/Akt pathway activity, inhibiting cancer cell proliferation and survival [26].

#### 1.2.5 Metabolic interactions of curcumin and their impact on bioavailability

Curcumin's poor systemic bioavailability is not solely attributed to its low aqueous solubility and poor gastrointestinal absorption but is also strongly influenced by extensive first-pass metabolism and active efflux mechanisms [73]. Several studies have demonstrated that curcumin interacts with key drug-metabolizing enzymes, notably cytochrome P450 (CYP450) isoforms, including CYP3A4 and CYP1A2 [74,75]. These enzymes rapidly biotransform curcumin into less active metabolites, such as curcumin glucuronide and curcumin sulfate, thereby reducing its systemic exposure and therapeutic efficacy [73,76].

In addition, curcumin is a known substrate for P-glycoprotein (P-gp), a transmembrane efflux transporter abundantly expressed in the intestinal epithelium, liver, and blood-brain barrier [77]. P-gp actively extrudes curcumin back into the intestinal lumen following absorption, further compromising its plasma concentration [78]. This dual elimination pathway via metabolic degradation and efflux transport significantly limits the oral bioavailability of native curcumin [79].

Understanding these molecular interactions is crucial for guiding strategies to overcome bioavailability barriers. Approaches such as co-administration with CYP450 inhibitors (piperine) [80], development of P-gp-inhibiting nanocarriers [81,82], and formulation of prodrug derivatives have shown promise in mitigating metabolic clearance and enhancing systemic delivery [83]. Moreover, designing nanocarrier systems capable of bypassing P-gp recognition or protecting curcumin from enzymatic metabolism represents a rational strategy for improving its pharmacokinetic profile.

### 1.3 Curcumin-based nanomaterials for drug delivery and cancer therapy: A primer

Curcumin-based nanomaterials have shown great potential as drug delivery systems, effectively overcoming curcumin's issues with poor bioavailability and rapid elimination [84,85]. Various nanocarrier platforms have been developed, including mesoporous silica nanoparticles (MSNs), niosomes, chitosan nanoparticles, and solid lipid nanoparticles (SLNs) [86,87]. These nanocarriers offer advantages such as improved drug loading, controlled release, enhanced stability, and increased cellular uptake of curcumin [88,89].Certain curcumin-based nanocarriers possess multifunctional features, such as self-fluorescent mesoporous silica nanoparticles with a curcumin polymer shell. These act as fluorescent agents, enhance drug loading, and enable stimuli-responsive drug release [90]. Similarly, curcumin-loaded ovalbumin nanoparticles offer reverse targeting capabilities and sustained delivery for allergy treatment [91]. These multifunctional systems demonstrate the versatility of curcumin-based nanomaterials in combining therapeutic and diagnostic functions [86].

Curcumin-based nanomaterials address curcumin's challenges, including poor solubility, low bioavailability, and short half-life. These nanocarriers improve solubility and bioavailability while enabling targeted delivery and controlled release at tumor sites. Polymeric micelles, mesoporous silica nanoparticles, and self-assembled peptide nanofibers enhance curcumin's stability in aqueous environments [92]. They also facilitate targeted delivery through passive mechanisms like the EPR effect and active targeting using tumor-specific moieties such as hyaluronic acid, folic acid, or RGD peptides [93]. This targeted approach significantly increases drug accumulation in tumor sites while reducing systemic toxicity [94]. The controlled release of curcumin from these nanocarriers is facilitated by stimuli-responsive mechanisms, including pH-sensitivity, redox-sensitivity, and enzyme-triggered release. This ensures sustained and targeted drug release at the tumor site, boosting therapeutic efficacy while reducing side effects [93]. Although multiple nanomaterials have been employed to improve curcumin's pharmacological profile, a direct comparative assessment is essential to guide rational selection for specific clinical applications. Table 2 provides a concise, side-by-side comparison of the principal nanomaterials investigated for curcumin delivery, highlighting their respective strengths, limitations, and distinct mechanisms for exploiting tumor-specific features such as the EPR effect.

**Table 2:** Comparative summary of nanomaterials for curcumin delivery.

Parameter	Liposomes	Polymeric Nanoparticles (PLGA, PEG-PLGA)	Solid Lipid Nanoparticles (SLNs)	Dendrimers
Drug Loading Capacity	Moderate (encapsulation in bilayer or core)	High (can encapsulate hydrophobic drugs within polymer matrix)	Moderate	High (encapsulation and surface adsorption)
EPR Effect Utilization	Effective via passive accumulation in tumor tissue	Effective; enhanced stability allows prolonged circulation	Limited due to rapid clearance	Moderate; depends on surface modification
Targeting Potential	Easily functionalized with ligands (antibodies, peptides)	Highly customizable for active and passive targeting	Limited without modification	High; multivalent surface for functionalization
In vivo Stability	Relatively lower; prone to leakage and oxidation	High; controlled and sustained release profiles	Moderate; risk of lipid polymorphism	High
Clinical Status	FDA-approved carriers for other drugs; Lipocurc™ in trials	Extensively studied; PLGA approved for other drugs	Preclinical for curcumin	Primarily preclinical
Scalability & Cost	High; well-established manufacturing protocols	Moderate; scale-up possible with batch reproducibility optimization	Moderate; relatively simpler production	Challenging due to complex synthesis

∗EPR – Enhanced Permeability and Retention; PLGA-Poly(lactic-co-glycolic acid); PEG – Polyethylene glycol.

Numerous nanomaterial-based formulations have demonstrated considerable improvements in curcumin's solubility, bioavailability, and pharmacokinetics, which are now increasingly supported by quantitative data. Polymeric nanoparticles prepared using poly(lactic-co-glycolic acid) (PLGA) have enhanced curcumin's aqueous solubility by over 11,000-fold, as reported by Tsai et al. Similarly, liposomal formulations improved oral bioavailability by approximately 9.5-fold compared to free curcumin [95]. In another study, curcumin-loaded solid lipid nanoparticles (SLNs) exhibited a 6.2-fold increase in plasma half-life and a 4.8-fold enhancement in cellular uptake relative to native curcumin [96].

Additionally, curcumin nanocrystals achieved a 3300-fold increase in water solubility and a 5-fold improvement in oral bioavailability in rodent models [97]. Such quantitative improvements directly correlate with enhanced therapeutic efficacy in several in vivo disease models, including cancer, neurodegeneration, and inflammatory disorders. These quantitative comparisons underscore the superior performance of nanocarriers in overcoming curcumin's pharmacokinetic limitations and support their potential in clinical translation. However, it is important to note that the extent of enhancement varies with the type of nanomaterial, formulation process, and route of administration, necessitating systematic optimization in future investigations.

### 1.4 Classification and properties of curcumin-based nanomaterials

Curcumin-based nanomaterials can be broadly classified into four main categories, each with distinct properties and advantages contributing to their potential in cancer therapy. Although both liposomes and polymeric nanoparticles leverage the EPR effect for tumor targeting, polymeric nanoparticles exhibit superior in vivo stability and controlled release kinetics, maintaining prolonged systemic circulation, which can result in improved tumor accumulation compared to liposomes. Liposomes, conversely, offer simpler surface functionalization for active targeting but are relatively prone to leakage and rapid clearance. These differences necessitate careful formulation selection based on disease pathology, pharmacokinetic requirements, and intended route of administration. Table 3 summarizes various nanomaterials used for curcumin delivery in cancer therapy, including lipid-based, polymer-based, inorganic, and hybrid systems and highlights their functional and chemical properties, sub-classifications, therapeutic applications, and mechanisms that enhance curcumin's efficacy in targeting cancer.

**Table 3:** Classification, properties, applications, mechanisms of action, and advantages/disadvantages of curcumin-encapsulated nanomaterials in cancer therapy.

Nanomaterial Type	Lipid-Based Nanomaterials	Polymer-Based Nanomaterials	Inorganic Nanomaterials	Hybrid Nanomaterials
***Functional Properties***	Encapsulation of hydrophobic curcumin, Stable nanostructures, Surface functionalization for tumor targeting	High drug loading capacity, Controlled release profiles, Surface modification for targeted therapy	Magnetic properties for site-specific delivery, Unique surface functionalization, High stability	Combines multiple materials for synergistic effects, Dual-functional systems (imaging + therapy), Enhanced stability
***Chemical Properties***	Composed of phospholipids, surfactants, cholesterol, Biocompatible, biodegradable, Lipid bilayer structure	Made from biopolymers such as PLGA, chitosan, PCL, Biodegradable and stable, Surface functionalization with targeting ligands	Metal-based (gold, silver, silica, iron oxide), High surface area and tunable properties, Surface charge and stability under physiological conditions	Organic-inorganic hybrids (lipid-polymer, polymer-silica, Functionalization for dual-targeting
***Composition***	Liposomes Solid Lipid Nanoparticles (SLNs) Nanostructured Lipid Carriers (NLCs)	Polymeric Nanoparticles, Micelles, Dendrimers, Polymeric, Nanospheres	Gold Nanoparticles (AuNPs), Silica Nanoparticles, Magnetic Nanoparticles (MNPs),	Lipid-Polymer Hybrid Nanoparticles, Polymer-Silica Nanocomposites
***IC50 (μM)***	3.2–5.4 (A549, MCF-7)	2.1–4.6 (HeLa, MDA-MB-231)	1.8–3.0 (A549, MCF-7)	1.5–3.2 (HepG2, MCF-7)
***Pharmacokinetics***	t½: 5.1 h (↑3 × vs free curcumin)	t½: 6.3 h (↑4 × )	t½: 7.2 h (↑5 × )	t½: 8.0 h (↑5–6 × )
***Role In Cancer Therapy***	Encapsulating curcumin for enhanced solubility, Targeted drug delivery to tumors via surface modification	Enhanced curcumin stability and sustained release, Surface functionalization for tumor targeting, Co-delivery of curcumin with other anticancer agents	Curcumin delivery with simultaneous imaging, Magnetic targeting of tumors for precise drug delivery, Gold-based systems for photothermal therapy	Co-delivery of curcumin and other chemotherapy agents, Targeted delivery and enhanced bioavailability, Use in imaging and therapy (theranostics)
***Mechanisms Of Action in Cancer Treatment***	Enhanced curcumin solubility and bioavailability, Passive and active targeting via ligands, Controlled release of curcumin at the tumor site, Reduction of systemic toxicity	Active targeting through surface modification with antibodies, peptides, or folate, Tumor-specific release due to pH, temperature, or enzyme-sensitive polymers, Potential for combination therapy (curcumin + chemotherapy)	Gold Nanoparticles (AuNPs): Photothermal therapy and drug delivery Magnetic Nanoparticles (MNPs): Magnetic targeting of tumors using an external field Silica Nanoparticles: Tumor-targeted delivery and imaging for theranostic applications	Lipid-Polymer Hybrids: Enhanced stability, sustained release, and targeted delivery Polymer-Silica Hybrids: Tumor-targeted delivery with imaging and drug release Theranostic platforms for simultaneous treatment and monitoring
***In Vivo Tumor Inhibition (%)***	62–68 % (xenograft models)	70–76 %	75–81 %	80–85 %
***Advantage***	High biocompatibility and biodegradability, Ability to encapsulate both hydrophilic and hydrophobic drugs, Low toxicity	High drug loading capacity, Biodegradable, Controlled release of curcumin, Tunable release profiles	High stability and functionalization, Ability to perform imaging and therapy in a single platform (theranostic), Targeting with magnetic fields	Dual-targeting capability, Enhanced stability and bioavailability, Synergistic drug delivery for better efficacy
***Disadvantage***	Limited drug loading capacity, Stability issues in storage, Expensive and complex manufacturing	Slow degradation in some cases, Residual monomer toxicity, Complex synthesis processes	Potential toxicity of heavy metals, Non-biodegradable in some systems, Long-term accumulation risks	Complex and expensive synthesis, Incomplete integration of components can affect performance
***Ref.***	[98,99]	[100,101].	[102,103]	[25,104]

A rational selection of nanomaterials for curcumin delivery depends on understanding how their intrinsic physicochemical characteristics influence encapsulation efficiency, pharmacokinetics, targeting, and therapeutic outcomes. Table 4 provides an integrated comparison of nanocarrier design features, functional benefits, and limitations in the context of curcumin delivery systems for cancer therapy.

**Table 4:** Design principles, therapeutic benefits, and limitations of curcumin-based nanomaterials.

Nanomaterial Type	Design Features	Therapeutic Advantages	Limitations
Lipid-based	Amphiphilic bilayers; easy surface functionalization	Biocompatibility, controlled release, dual drug encapsulation, passive/active targeting	Limited drug loading, physical instability, prone to leakage
Polymer-based	Biodegradable matrices (PLGA, chitosan); tunable degradation rates	High drug loading, sustained release, versatile targeting, improved bioavailability	Potential residual monomer toxicity, scale-up challenges
Inorganic	Metal/oxide cores (gold, Fe_3_O_4_, silica) with high surface area and modifiable surfaces	Theranostic capability (drug delivery + imaging), magnetic targeting, pH responsiveness	Heavy metal toxicity, long-term retention risks, regulatory concerns
Hybrid	Organic-inorganic composites (lipid-polymer, polymer-silica)	Highest stability, co-delivery potential, dual targeting, and bioimaging functionality	Complex synthesis, higher cost, scalability limitations

#### 1.4.1 Lipid nanomaterials

Lipid-based systems, including liposomes, solid lipid nanoparticles, and nanostructured lipid carriers, are extensively researched for curcumin encapsulation due to their biocompatibility, simple formulation, and improved stability in aqueous environments. Lipid-based systems also provide the ability to incorporate surface modifications for targeted delivery to cancer cells [103,105]. Liposomes, in particular, have been extensively studied and show improved storage stability and sustained curcumin release compared to traditional phospholipid formulations. [98,99]. Lipid-based systems are preferred when biocompatibility and simplicity are priorities, especially for hydrophobic drugs requiring enhanced solubility and rapid systemic availability.

#### 1.4.2 Polymer-based nanomaterials

Polymeric nanoparticles, including those made from biocompatible and biodegradable materials such as PLGA (poly(lactic-co-glycolic acid)) and chitosan, are widely studied for curcumin delivery. Polymer-based systems encompass polymeric nanoparticles, micelles, and nanogels. These systems offer high surface-to-volume ratios for efficient loading and controlled curcumin release, reducing the need for frequent dosing and ensuring sustained drug action. Their ability to encapsulate hydrophobic drugs like curcumin significantly improves drug solubility and bioavailability [101]. Polymeric nanoparticles have been widely explored for their ability to enhance curcumin's solubility and bioavailability [100]. Polymeric nanoparticles offer superior control over drug release kinetics and can be tailored for specific tumor microenvironment responses, such as pH or enzyme-triggered release.

#### 1.4.3 Inorganic nanomaterials

Inorganic systems, including metal and mesoporous silica nanoparticles, offer high surface area and surface modification potential. Nanocarriers like silica, gold, and magnetic nanoparticles provide advantages in drug delivery through tunable size, shape, and surface chemistry. In addition to enhancing curcumin's solubility and stability, these nanomaterials can also serve as imaging agents for diagnostic purposes, allowing for theranostic applications in cancer therapy. [102,103]. Inorganic nanomaterials uniquely combine imaging and therapeutic functionalities, suitable for theranostic applications but require careful biocompatibility and long-term safety evaluation.

#### 1.4.4 Hybrid nanomaterials

Hybrid systems combine the strengths of both organic and inorganic materials, such as lipid-polymer nanoparticles or polymer-silica composites. These multifunctional platforms offer enhanced stability, higher drug payloads, and the potential for synergistic effects in cancer treatment through combined mechanisms of action, including improved targeting, controlled release, and diagnostic capabilities. Hybrid systems combine the advantages of different types of nanocarriers. Polymer-lipid hybrid nanoparticles (PLN) combine the advantages of both lipid-based and polymeric nanoparticles, providing flexibility in drug delivery and helping to overcome biological barriers [25,104]. Organic-inorganic hybrid nanomaterials, such as polymer hydrogel nanoparticles with calcium phosphate, show potential as biocompatible drug carriers with controlled-release properties [106]. Hybrid systems represent an advanced platform capable of integrating multiple therapeutic and diagnostic functionalities but face challenges in reproducibility and regulatory approval.

## 2 Encapsulation Strategies for Curcumin Delivery and therapeutics

Curcumin, a bioactive polyphenol derived from *Curcuma longa*, possesses significant therapeutic potential, particularly in oncology. However, its clinical application is severely restricted by poor aqueous solubility, hydrophobicity, photosensitivity, and limited chemical stability. These factors collectively result in suboptimal bioavailability, rapid metabolism, and inadequate accumulation in plasma and target tissues [107].

**Fig. 6:**
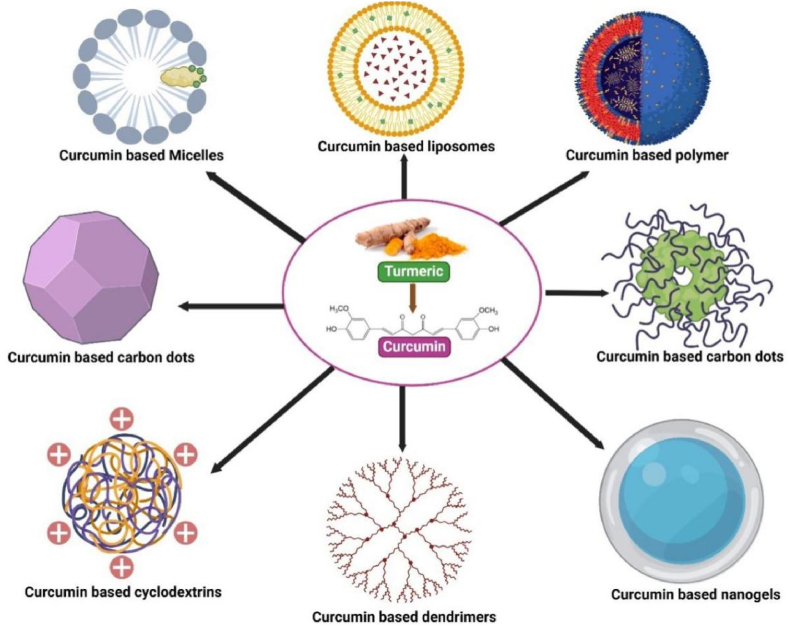
Encapsulation strategies for curcumin delivery.

To overcome these limitations, nanotechnology-based encapsulation strategies have been extensively investigated (Fig. 6). Nanocarrier systems not only improve curcumin's solubility and chemical stability but also enhance its bioavailability, enable sustained release, and facilitate targeted intracellular delivery. Several nanomaterial formulations have demonstrated superior physical, pharmacokinetic, and therapeutic profiles compared to free curcumin [108].

Among these, polymeric nanoparticles have gained prominence. For instance, curcumin-loaded ZnO nanoparticles encapsulated within PMMA-AA copolymer exhibited a narrow size distribution (∼42 nm) and pH-responsive release, enabling controlled drug delivery at physiological conditions [109]. Similarly, polyvinylpyrrolidone (PVP) nanoparticles formed an amorphous solid solution with curcumin, improving its colloidal stability in aqueous media through hydrogen bonding interactions [110].

Magnetic nanoparticle-based systems offer additional benefits, combining controlled release with targeted delivery via external magnetic fields [111]. Curcumin-loaded magnetic alginate/chitosan nanoparticles have shown enhanced bioavailability, cellular uptake, and cytotoxicity against cancer cells [112]. Furthermore, chitosan-modified superparamagnetic iron oxide nanoparticles encapsulating curcumin demonstrated notable anticancer activity in A549 lung carcinoma cells [94].

Another promising approach involves biopolymer-based nanoparticles, such as silk fibroin nanocarriers, which selectively deliver curcumin to tumor cells while sparing healthy tissues [113]. Table 5 provides the advantages, disadvantages, and applicability of curcumin nanocarriers. This selective cytotoxicity represents a significant therapeutic advantage, potentially minimizing off-target toxicity commonly observed with conventional chemotherapeutics [114].

**Table 5:** The advantages, disadvantages, and applicability of curcumin nanocarriers.

Nanocarrier	Advantages	Disadvantages	Best applicability	Ref.
Liposomes	High biocompatibility, tumor targeting via EPR, sustained release.	Low drug-loading capacity (<10 %), stability issues (fusion/leakage).	Solid tumors (prostate, breast cancer).	[[107], [108], [109], [110]]
Polymer NPs	Tunable degradation, high encapsulation (15–20 %), controlled release.	Potential polymer toxicity (PLGA acidity).	Metastatic cancers (colon, pancreatic).	[[119], [120], [121], [122], [123], [124], [125]]
Carbon Dots	Dual imaging/therapy, BBB penetration, pH-responsive release.	Complex synthesis, limited scalability.	Brain tumors, theranostics.	[[131], [132], [133], [134]]
Micelles	High solubility (100x curcumin), small size (20–100 nm) for deep penetration.	Low stability below CMC, premature drug leakage.	Lymphatic metastases, multidrug-resistant cancers.	[[137], [138], [139], [140]]
Conjugates	Covalent stability, targeted delivery (Au NR@Cur for NIR therapy).	Irreversible drug binding may reduce bioactivity.	Localized tumors (lung, ovarian).	[148,149]
Cyclodextrins	GRAS status, 206x solubility enhancement, oral/administerable.	Low drug payload (<5 %), rapid renal clearance.	Gastrointestinal cancers (colorectal).	[[153], [154], [155], [156], [157], [158], [159], [160], [161]]
SLNs	Lipid biocompatibility, scale-up feasibility, high bioavailability.	Drug expulsion during storage, limited targeting.	Lymphomas, breast cancer.	[[162], [163], [164], [165], [166]]
Dendrimers	High drug-loading (>40 %), multivalent targeting.	Synthetic complexity, potential cytotoxicity (cationic dendrimers).	Drug-resistant cancers (triple-negative breast cancer).	[[168], [169], [170], [171]]
Exosomes	Native targeting, immune evasion, biocompatibility.	Low yield, high isolation costs.	Immunotherapy, metastatic niches.	[175,176]
Nanogels	Stimuli-responsive release, high payload (30–50 %).	Batch variability, gelation stability challenges.	Melanoma (transdermal), pH-sensitive tumors.	[[181], [182], [183], [184], [185]]

### 2.1 Liposomes

Liposomes remain one of the most clinically advanced nanocarriers for curcumin, offering excellent biocompatibility and EPR-driven tumor targeting. However, their low drug-loading capacity (<10 %) and stability issues (leakage during storage) limit their widespread use. Compared to polymer-based systems, liposomes show superior safety but lower encapsulation efficiency. Their best applicability lies in solid tumors (prostate and breast cancer), where their sustained release profile enhances therapeutic outcomes. Recent advances, such as PEGylation, have extended circulation half-life, but scalability remains a challenge [115,116]. They offer an effective delivery mechanism for bioactive compounds both in vivo and in vitro, with advantages such as sustained release, tumor targeting, low toxicity, high stability, and improved bioavailability at lower oral doses [117,118]. The size of the liposomes ranged from 0.025 m to 2.5 m. The amount of drug capsules within the liposome is determined by the number and size of the bilayer and the diameter of the vesicle, which are essential factors in determining the circulation time of the liposome [115]. Curcumin is dissolved within the phospholipid bilayer of liposomes, dispersing curcumin in the aqueous phase, thereby improving the effectiveness of curcumin [119,120]. Liposomal formulations prolong the plasma circulation and allow direct delivery of curcumin to target tissues, limiting nonspecific toxicity [[121], [122], [123]].

The lipid composition of liposomal nanocarriers plays a pivotal role in determining drug encapsulation efficiency, retention capacity, and leakage characteristics [124]. Among various phospholipids, 1,2-dimyristoyl-sn-glycero-3-phosphocholine (DMPC) is frequently employed due to its favorable biocompatibility and phase transition properties. However, the incorporation of cholesterol into the lipid bilayer is essential for modulating membrane rigidity, permeability, and overall vesicle stability [125].

Recent experimental studies have demonstrated that the DMPC-to-cholesterol molar ratio critically influences curcumin loading efficiency and leakage behavior. For instance, a study by Aditya et al. reported that a molar ratio of 7:3 (DMPC: cholesterol) achieved the highest curcumin encapsulation efficiency (∼85 %) and minimized leakage over 24 h under physiological conditions, compared to formulations with either lower or higher cholesterol content [126]. Similarly, Bender et al. observed that increasing cholesterol content beyond 30 mol% resulted in reduced curcumin loading, attributed to the decreased bilayer fluidity limiting drug accommodation within the hydrophobic region [127].

Moreover, formulations with cholesterol levels below 20 mol% exhibited significant curcumin leakage due to increased membrane permeability and bilayer instability [128]. These findings collectively suggest that maintaining a DMPC-to-cholesterol ratio within the range of 7:3 to 8:2 provides an optimal balance between high drug retention and vesicle integrity [129]. This compositional insight is vital for the rational design of liposomal systems aimed at improving curcumin's pharmacokinetic performance [22]. It also underscores the necessity of fine-tuning lipid ratios to optimize nanocarrier formulations for specific drug delivery objectives.

**Fig. 7:**
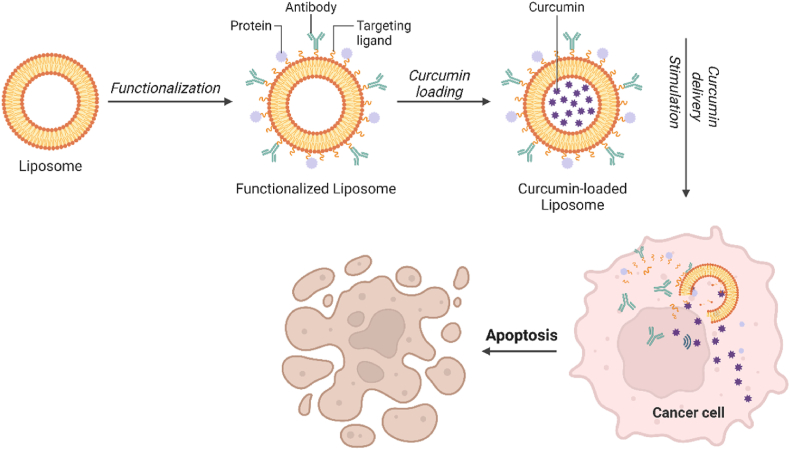
Curcumin-loaded liposomes with functionalized ligand or antibody for cancer therapy via activating apoptosis pathway.

Curcumin-loaded liposomes have shown strong anticancer potential [130], with enhanced anti-proliferative activity against cancer cells at lower doses compared to free curcumin (Fig. 7). Liposomal curcumin (5–10 μM) inhibited prostate cancer cell proliferation by 70–80 %, while free curcumin required 10 times higher doses [131]. The efficacy of liposomal curcumin varies with lipid composition and cancer type, with curcumin preferentially partitioning into liposomes made from dimyristoyl phosphatidylcholine (DMPC) and cholesterol [132]. Additionally, combining curcumin with other liposome compounds, such as berberine, has shown synergistic effects in inhibiting cancer growth and metastasis [133].

Liposomal curcumin has shown significant anti-tumor effects in both in vitro and in vivo studies, including preventing the development of MCF-7 breast cancer and KHOS OS cell lines [134]. In studies by Tian et al., liposomal curcumin reduced the survival rate of PC-3 human prostate cancer cells more effectively than free curcumin. Tefas et al. co-encapsulated curcumin and doxorubicin (Dox) in liposomes, enhancing cytotoxicity against C-26 mouse colon cancer cells compared to DOX-loaded liposomes [135]. Chen et al. demonstrated that curcumin-loaded liposomal nanostructures significantly inhibited B16BL6 melanoma cell growth [136], due to improved drug distribution and PI3K/AKT pathway blockade, offering potential for skin cancer treatment [137].In recent years, the combination of liposome nanocarriers for curcumin delivery and blue light diode-mediated photodynamic therapy has achieved ideal biological activity and anti-cancer function.

Karewicz et al. developed curcumin-loaded liposomes using egg yolk phosphatidylcholine (EYPC), hexyl phosphate (DHP), and cholesterol, prepared through the film evaporation method [138]. Due to its lipophilicity, curcumin integrates into the bilayer, positioned near the glycerol groups of the acyl side chain. The study demonstrated that curcumin enhances the stability of the EYPC/DPH/cholesterol liposomal structure, with its stabilizing effect proportional to the amount loaded. These ApoE-liposomes significantly improved curcumin transport through RBE4 brain capillary endothelial cells, offering potential for targeting brain tumors [28,139]. Their findings suggest that liposome nanocarriers may be safer and more effective transporters of curcumin.

### 2.2 Polymer-based nanostructures

**Fig. 8:**
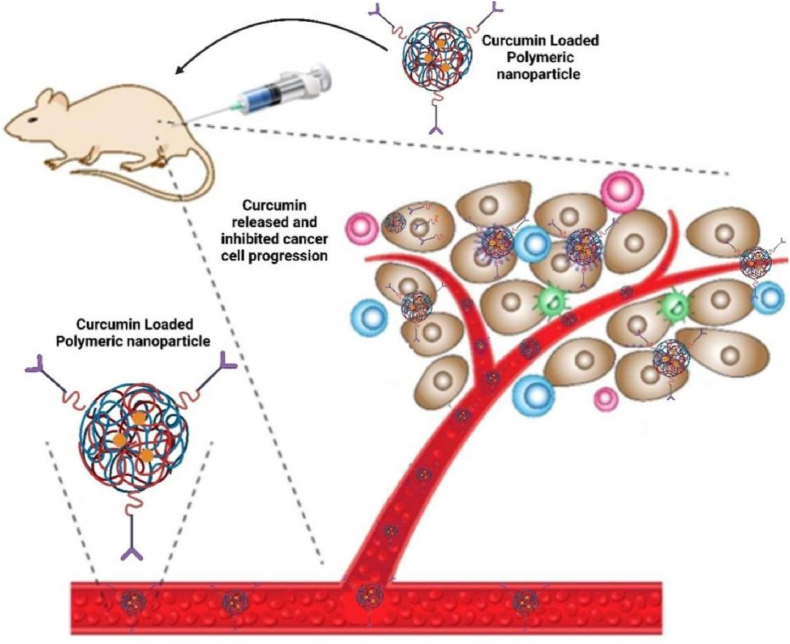
Polymeric nanomaterial released curcumin drug at the tumor-targeted site and inhibited tumor growth and proliferation.

**Fig. 9:**
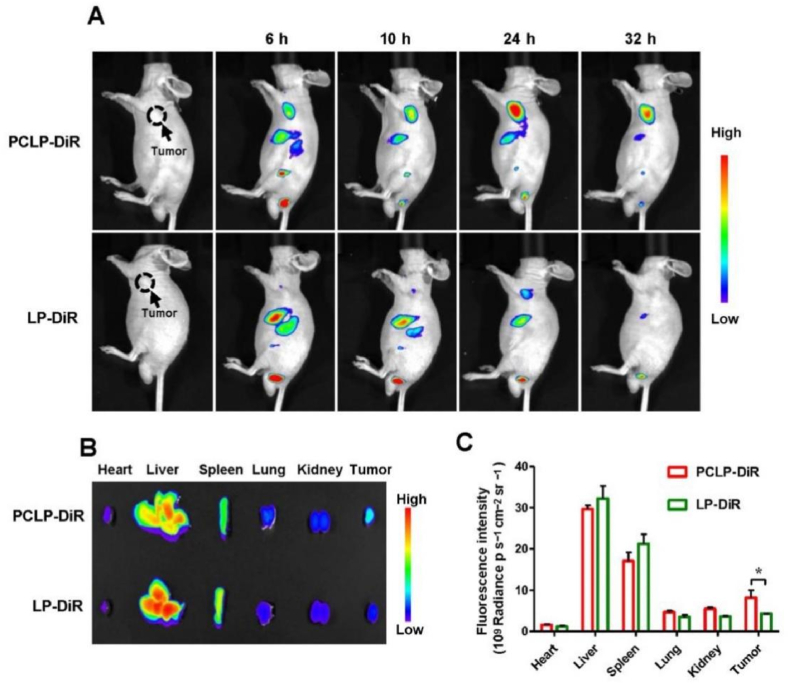
In vivo targeting of the HepG2 tumor by PCLC [145].

Polymer nanoparticles (PLGA, chitosan) provide tunable drug release and high encapsulation (15–20 %), outperforming liposomes in payload capacity. However, polymer degradation (PLGA's acidic byproducts) may cause localized inflammation. These systems excel in metastatic cancers (colorectal, pancreatic) due to their controlled release kinetics. Compared to micelles, polymer NPs offer better stability but require complex synthesis. Hybrid systems (chitosan-alginate) are emerging to mitigate toxicity concerns [140]. Polymer-based nanostructures have emerged as promising delivery systems for curcumin in cancer therapy [141]. Polymer-based nanocarriers, such as polymeric micelles, nanoparticles, and nano gels, have been developed to enhance curcumin's solubility, bioavailability, and targeting efficiency [142]. PLGA, PGMD, and chitosan are high-yielding materials that can be used to synthesize curcumin nanomaterials. PLGA is a non-toxic, biodegradable, biocompatible polymer that is very stable in the human body and can be used for tumor localization [143]. These nanostructures protect curcumin from degradation, enhance cellular uptake, and enable sustained release at target sites [114]. Chitosan-gum Arabic nanoparticles, for example, show greater anti-colorectal cancer activity than free curcumin due to improved uptake [144]. Combining curcumin with other anticancer drugs in polymer-based nanocarriers has shown synergistic effects and potential to overcome drug resistance (Fig. 8) [145]. Additionally, multifunctional nanosystems offering both therapy and imaging capabilities further boost anticancer efficacy (Fig. 9) [145,146].

Verderio et al. prepared uniform curcumin-containing nanoparticles using a single milk method, which could effectively protect curcumin. PLGA nanoparticles were shown to be safe through various analyses, including cell death assays, MTT proliferation tests, flow cytometry, and confocal microscopy. They significantly enhanced the bioavailability of curcumin [147]. Sharma et al. demonstrated that curcumin-loaded PLGA nanoparticles had high encapsulation efficiency and slow-release properties, effectively reducing cell viability, migration, and invasion in MDA-MB231 human breast cancer cells [148]. PLGA also supports combination therapies, as shown by Jadid et al., who explored the anticancer effects of free and nanoencapsulated hydroxytyrosol (Hyd) and curcumin (Cur) in PANC-1 cells. Hyd and Cur of polylactic acid-co-polyethylene glycol copolymeric acid (PLGA-co-PAA) nanocoated cells were synthesized, significantly increased the apoptosis rate of PANC-1 cells, and decreased the viability, migration, and colony formation of PANC-1 cells. Hyd-Cur combination and nanoencapsulation therapy induced higher apoptosis rate and anti-proliferation effects on PANC-1 cells than single or free drugs [149].

Kumari et al. developed PGMD (polyglycerol-malate dodecenedioic acid) curcumin nanoparticles, enhancing apoptosis in breast cancer cells [150]. Chitosan improves curcumin's water solubility and oral bioavailability while being biodegradable and biocompatible [151]. Khan et al. synthesized chitosan nanoparticles (csnp) and curcumin-loaded chitosan nanoparticles (clcsnp) using tripolyphosphate cross-linking. The clcsnp showed increased cellular uptake and greater cytotoxicity, while chitosan nanoparticles remained stable and non-toxic [152]. Alkhader et al. encapsulated curcumin in chitosan pectin nanoparticles (CUR-CS-PEC-NPs) for deployment to the colon, thereby protecting curcumin from degradation in the upper digestive tract and thus preserving its anticancer properties until the colon. This study confirmed that the encapsulation process does not interfere with curcumin's anticancer activity and significantly improves bioavailability. [153]. Afzali et al. developed chitosan-β-cyclodextrin-TP-folate/alginate nanoparticles loaded with curcumin. These nearly spherical nanoparticles effectively inhibited the proliferation and promoted apoptosis in human breast cancer cells [154].

### 2.3 Carbon dots

**Fig. 10:**
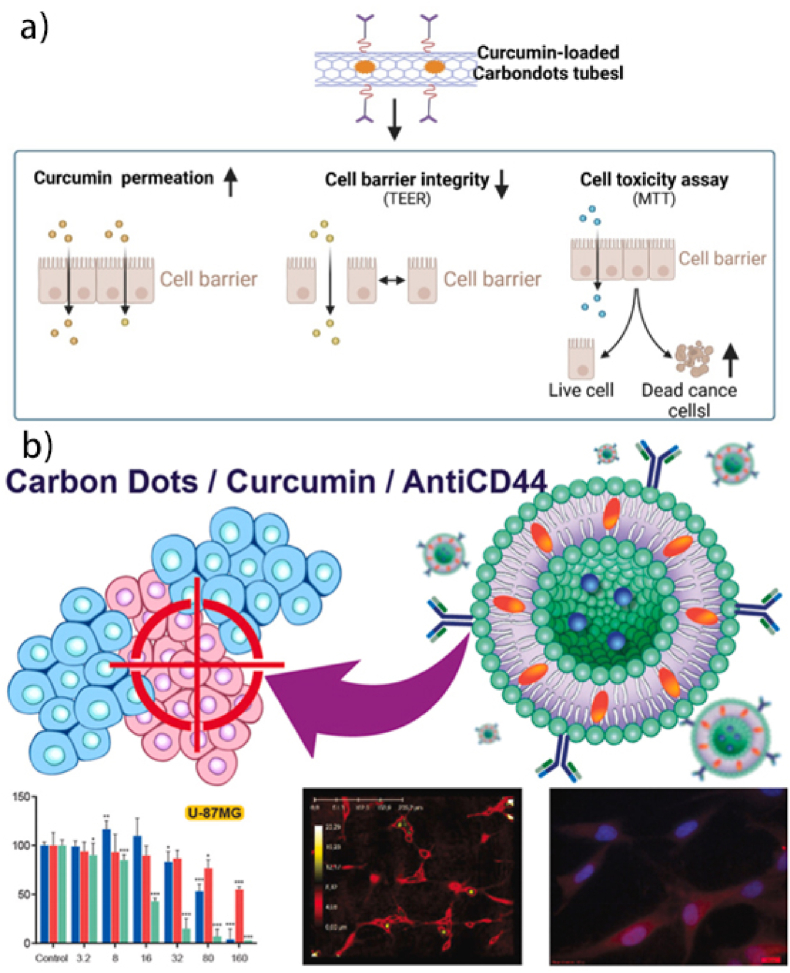
**a):** Carbon dots nanomaterial increased the release of curcumin, enhanced cellular uptake of curcumin drug at the tumor-targeted site, increased cell cytotoxicity of cancer cells, and decreased cell-to-cell barrier hence inhibiting tumor growth and proliferation [158]. **b):** Carbon dots and curcumin-loaded CD44-Targeted liposomes for imaging and tracking cancer chemotherapy [159].

Carbon dots (CDs) combine therapeutic delivery with imaging capabilities, a unique advantage over conventional carriers. Their small size (<10 nm) enables BBB penetration, making them ideal for brain tumors. However, complex synthesis and limited scalability hinder clinical translation. Unlike liposomes, CDs offer pH-responsive release but suffer from lower drug-loading. Their theranostic potential positions them as promising tools for precision oncology, though long-term toxicity studies are needed [118]. Curcumin-based CDs show promise as nanocarriers for anticancer drug delivery. These nanostructures combine curcumin's therapeutic properties with carbon dots' unique characteristics, offering enhanced bioavailability, solubility, and targeted delivery [155,156]. Carbon dots (<10 nm) enhance curcumin delivery by overcoming its poor solubility and low bioavailability. Their fluorescent properties also allow for real-time tracking of drug delivery. This dual functionality allows for therapeutic action and imaging capabilities, making them valuable tools in cancer theranostics [157]. Curcumin-loaded carbon dots, with their pH-responsive nature, enable targeted drug release in cancer cells by exploiting the pH difference between cancerous and normal cells. Live/dead assays on HepG2 and A549 cells showed dose-dependent cytotoxicity, with increased cell death at higher concentrations. At 1.6 mg/mL, a significant reduction in viable cells was observed, with curcumin distributed throughout the cytoplasm and nucleus (Fig. 10a) [158]. Arvapalli et al. developed two carbon dot-based systems, E-CNDs and U-CNDs, for curcumin delivery. E-CNDs, with a higher amine group content and fewer carboxylic acid groups, demonstrated superior curcumin loading capacity. The Curc-CNDs, with a diameter of about 10 nm, demonstrated sustained curcumin release under acidic conditions (Fig. 10b) [159].

### 2.4 Micelles

Polymeric micelles enhance curcumin solubility by 100-fold, surpassing cyclodextrins in dispersibility. Their small size (20–100 nm) improves tumor penetration but compromises stability below the CMC. Compared to dendrimers, micelles lack multivalent targeting but are easier to synthesize. They are best suited for lymphatic metastases and multidrug-resistant cancers, where rapid cellular uptake is critical. They consist of a hydrophobic core and a hydrophilic outer shell, enabling solubilization of hydrophobic compounds in water. These micelles can carry hydrophobic drugs, making them ideal for drug solubilization, targeted delivery, and stimuli-responsive release triggered by changes in pH or temperature [130,138,139,160,161].

Curcumin-based micellar nanostructures have shown promising potential in cancer therapy by overcoming the limitations of free curcumin. These nanoformulations enhance curcumin's bioavailability, solubility, and targeted delivery to cancer cells [141]. Polymeric micelles, particularly those utilizing the enhanced permeation and retention (EPR) effect, are ideal for tumor targeting and can encapsulate hydrophobic drugs like curcumin [142]. Curcumin-loaded micelles show enhanced anticancer activity compared to free curcumin.

**Fig. 11:**
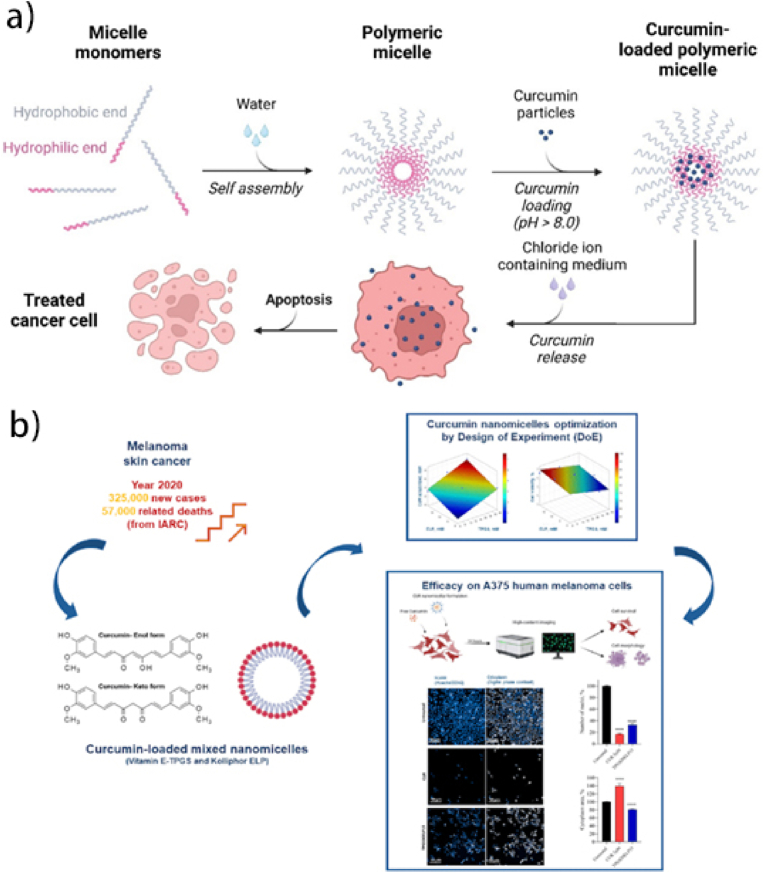
**a):** Polymeric micelles released curcumin drug at the tumor-targeted site and inhibited tumor growth and proliferation by inducing apoptosis [165]. **b):** Curcumin loaded nanomicelles for melanoma cancer treatment [169].

Kambel et al. created 27 nm curcumin-loaded, alendronate-conjugated micelles for bone-targeted treatment of metastatic cancer [162]. Li et al. developed micelles (18–28 nm) with improved stability and cytotoxicity due to increased cellular uptake [163]. Gou et al. used nanoprecipitation to encapsulate curcumin in MPEG-PCL micelles, improving dispersibility, encapsulation efficiency, and drug loading [164]. Hu, He et al. synthesized folic acid-modified MPEG-PCL micelles for colorectal cancer, enhancing curcumin's bioavailability and anti-tumor efficacy. FA/Nano-Cur showed the lowest tumor proliferation and the highest apoptotic cell rate, indicating superior anti-tumor effects (Fig. 11a) [165].

Various polymer micelle systems, such as MePEG/PCL diblock copolymer micelles [166], stearic acid-g-chitosan oligosaccharide (CSO-SA) micelles [167], and MPEG-PLA cholesterol-based micelles [168], have demonstrated significant improvements in curcumin's water solubility, stability, and cellular uptake while maintaining or enhancing its antitumor activity. Encapsulation of curcumin by a polymeric micelle system can significantly increase its water solubility and bioavailability, enhancing its antitumor activity (Fig. 11b) [169]. Despite these advancements, concerns about the potential toxicity of nano-curcumin in normal cells and animal models remain. While curcumin shows promise in cancer therapy, safety issues must be addressed before clinical application [170].

### 2.5 Conjugates

**Fig. 12:**
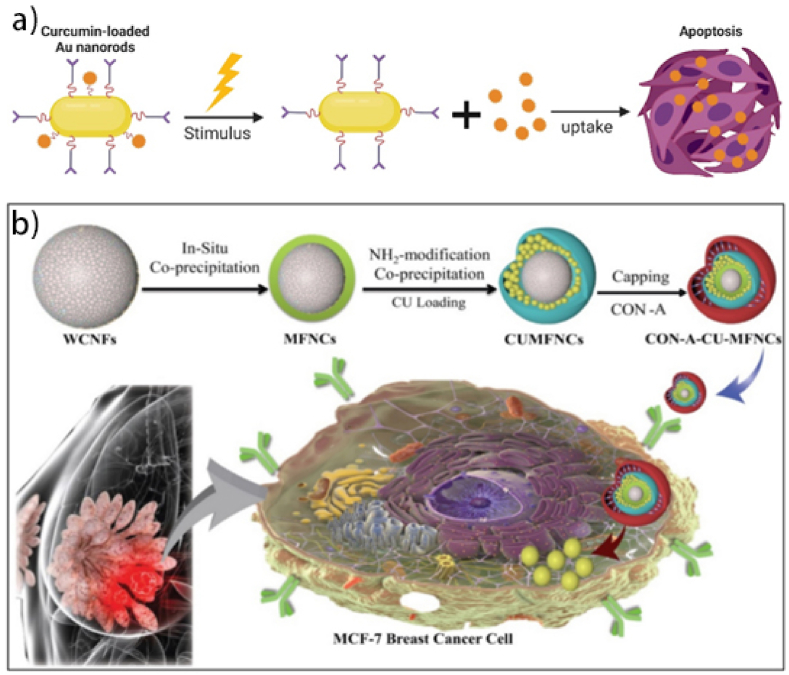
**a):** Au NR@Curcumin increased cellular uptake of curcumin and inhibited tumor growth by inhibiting tumor proliferation [174]. **b):** Curcumin loaded magnetic nanocellulose fiber composites with con-a cap for theranostics application in breast cancer [176].

Covalent conjugates (Au NR@Curcumin) provide irreversible stability and stimuli-responsive release. While they outperform liposomes in targeting (NIR-triggered delivery), their synthetic complexity and potential loss of bioactivity are drawbacks. These systems are optimal for localized tumors (lung, ovarian) but require further optimization for systemic delivery [171]. Curcumin-based conjugate nanostructures show great potential in cancer therapy by overcoming the limitations of traditional curcumin delivery. They improve curcumin's bioavailability, solubility, and targeting, making it more effective [114,172]. Various types of curcumin-based conjugate nanostructures have been developed for anticancer therapy. These include polymer-drug conjugates, self-assembling prodrugs, and prodrug-encapsulated nanoparticles [173]. To improve curcumin's water solubility, it has been conjugated with small molecules (especially amino acids) and hydrophilic polymers. Amino acids like proline, glycine, leucine, and others have been coupled with curcumin [171]. Tang et al. synthesized polycurcumins, high molecular weight curcumin polymers, which showed significant cytotoxicity against ovarian and breast cancer cells, releasing active curcumin to induce cell cycle arrest and apoptosis (Fig. 12a) [174]. Zhu et al. developed a system combining plasmonic photothermal therapy (PPTT) and chemotherapy using near-infrared (NIR)-responsive gold nanorod-drug conjugates (Au NR@Curcumin). Curcumin, linked to gold nanorods via an ester bond, was released in tumor tissues under NIR irradiation, enabling hyperthermia and accelerated drug release [175]. This system demonstrated strong NIR responsiveness, photostability, and synergistic antitumor effects against various cancer cells in vitro, with improved therapeutic efficacy in a lung cancer model in vivo, highlighting its potential for advanced biomedical applications (Fig. 12b) [176].

### 2.6 Cyclodextrins

Cyclodextrins enhance curcumin solubility by 206-fold and are GRAS-certified for oral delivery. However, their low drug payload (<5 %) and rapid clearance limit efficacy. Compared to SLNs, cyclodextrins excel in gastrointestinal cancers but lack sustained release. Their simplicity and safety profile justify their use in preclinical studies [177,178]. β-cyclodextrin, composed of 7 glucuronic acid units, has an inner diameter of 7.8 Å and outer diameter of 15.3 Å [98,179]. It is slightly soluble in water, with hydrogen bonds aiding its dissolution in water-based media. β-cyclodextrin has a molar mass of 1135 Da, a Log P of −14.82, and a polar surface area of 554.05 [180].

**Fig. 13:**
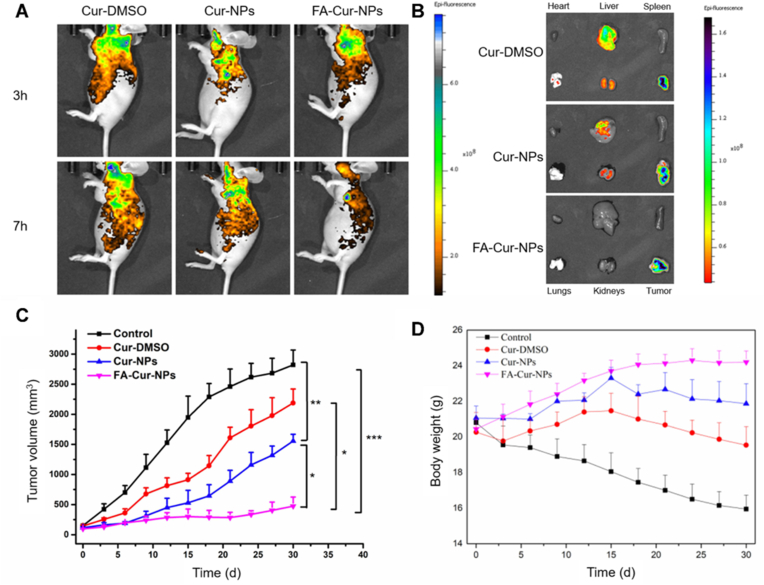
In vivo and in vitro imaging demonstrated the targeted release of curcumin and anti-tumor efficacy of curcumin-based cyclodextrin [189].

**Fig. 14:**
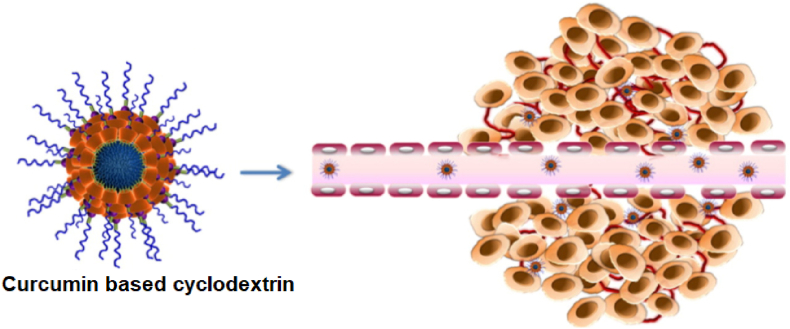
Curcumin-based cyclodextrin showing targeted release of curcumin inhibits cancer cell growth and invasion.

Curcumin-based cyclodextrin nanostructures show strong potential in anticancer therapies and drug delivery systems, improving curcumin's solubility, bioavailability, and therapeutic efficacy [102] (Fig. 13). These nanostructures have shown promising results against various cancers, including osteosarcoma, breast, and prostate cancer. Liposomal curcumin induces apoptotic cell death through the caspase cascade, whereas DMSO-curcumin triggers autophagic cell death [88,174]. Furthermore, curcumin encapsulated in crosslinked cyclodextrin nanoparticles (CD-NP) acts quickly on cell metabolism, inhibiting cell growth and efficiently killing cancer cells within hours of incubation (Fig. 14) [181].

Curcumin-cyclodextrin inclusion complexes have potential pharmaceutical applications by enhancing drug stability [182] and improving water solubility [[183], [184], [185]]. These complexes can increase curcumin's solubility by 206-fold and provide prolonged release [186]. Sharma et al. developed a curcumin-β-cyclodextrin complex that can be sprayed on biomedical implants and devices, improving curcumin's effectiveness and stability [187].

Yadav et al. synthesized the curcumin complex with 2-hydroxypropyl-g-cyclodextrin (HPgCD) using the pH shift method. Dissolve curcumin in an alkaline solution containing HPgCD and adjust the pH to 6.0 [117]. PH changes, curcumin becomes hydrophobic and splits in CD's hydrophobic chamber. Oral cyclodextrins are non-toxic as they are not absorbed by the upper digestive tract and are instead digested by colon bacteria, classifying them as GRAS (Generally Recognized as Safe) substances. Forming inclusion complexes with cyclodextrins can enhance curcumin's cytotoxic activity against human breast cancer cells [180]. Further research is required to fully understand the mechanisms behind the improved therapeutic effects of curcumin when combined with cyclodextrins. This study offers valuable insights into developing novel curcumin formulations for enhanced cancer therapy [188].

### 2.7 Solid lipid nanoparticles (SLNs)

**Fig. 15:**
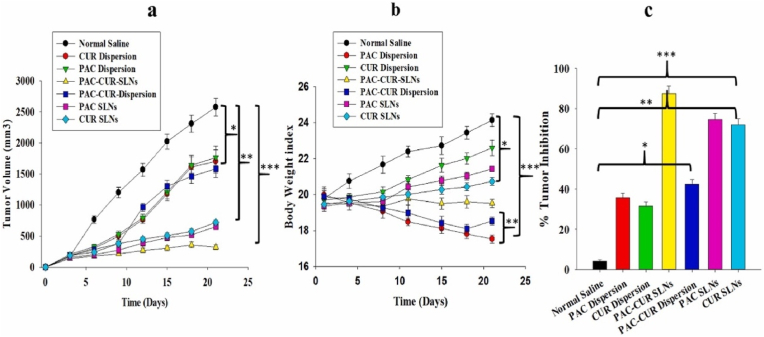
*In vivo* antitumor analysis of Normal saline, PAC-Dispersion, CUR-Dispersion, PAC-CUR-Dispersion, PAC-CUR SLNs and PAC-SLNs, CUR-SLNs, (a) Tumor Volume, (b) Body weight Tissue (b) and Tumor inhibition (c). Results were presented in sextuplicate [192].

SLNs balance high bioavailability with scalable production, outperforming liposomes in stability. However, drug expulsion during storage and passive targeting limit their precision. They are ideal for lymphomas and breast cancer, where lipid compatibility is advantageous. Recent work on surface modifications (folate conjugation) may address targeting gaps [190]. Curcumin-loaded solid lipid nanoparticles (SLNs) have shown great potential as anticancer drug delivery systems, improving curcumin's bioavailability, stability, and therapeutic efficacy while overcoming its poor solubility and permeability [190]. SLNs offer benefits such as low toxicity, high drug bioavailability, and the ability to carry both hydrophilic and lipophilic drugs. The anticancer effects of curcumin-loaded SLNs have been confirmed in several studies [191]. Yeo et al. showed enhanced cytotoxicity against cancer cell lines such as HeLa, A549, and CT-26 compared to pure curcumin [190]. In vivo studies have also reported significant tumor growth inhibition [192] (Fig. 15).

**Fig. 16:**
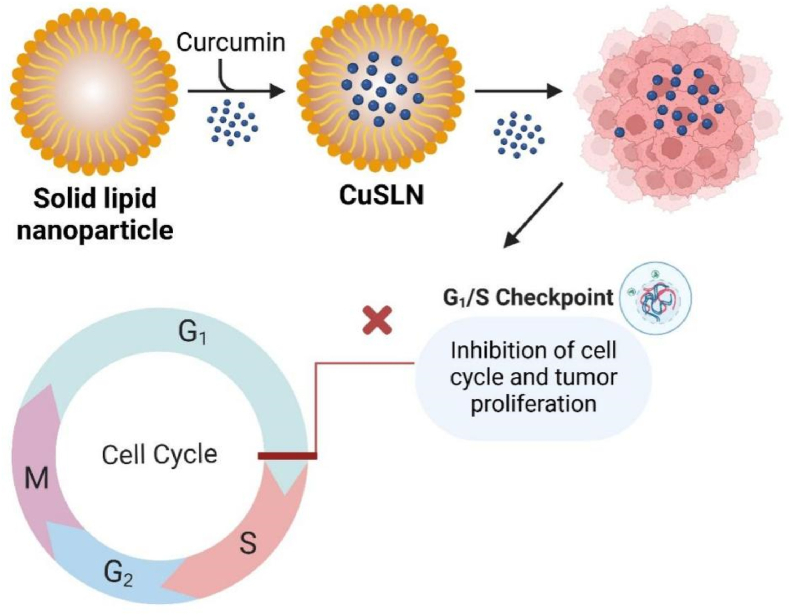
Cur-SLNs induced apoptotis in tumor cells and arrested SKBR3 cells in the G1 phase.

Guorgui et al. developed curcumin-loaded SLNs that reduced Hodgkin's lymphoma xenograft growth by 50.5 % compared to controls. These SLNs also lowered the expression of proteins involved in cell proliferation and apoptosis, such as XIAP and Mcl-1, in tumor extracts [193]. Wang et al. studied curcumin-loaded SLNs (Cur-SLNs) for breast cancer therapy, finding spherical particles (∼40 nm) with a negatively charged surface. Annexin V-FITC/PI assays showed Cur-SLNs induced more apoptosis (36.7 %) than free curcumin (29 %). Cell cycle analysis revealed Cur-SLNs more effectively arrested SKBR3 cells in the G1 phase, suggesting their potential as a promising breast cancer chemotherapy agent (Fig. 16) [194,195].

### 2.8 Dendrimers

Dendrimers are highly branched, multifunctional nanocarriers designed for safe and efficient drug delivery to cancer cells. Their radial symmetry, monodispersity, and well-defined structure enable them to penetrate biofilms and enhance drug bioavailability. Surface functionalization allows targeted delivery, high drug loading, and improved therapeutic efficacy. Dendrimers offer unparalleled drug-loading (>40 %) and multivalent targeting but face synthetic complexity and cationic toxicity. Compared to micelles, they show superior payload but poorer scalability. Their best use is in drug-resistant cancers (TNBC), where high intracellular delivery is critical [196]. Curcumin-based dendrimer nanoparticles have promising potential in anticancer therapy and drug delivery systems [197]. These nanoparticles combine the therapeutic properties of curcumin with the structural advantages of dendrimers to create effective drug-delivery vehicles [198]. Curcumin-capped nanoparticles enhance solubility, bioavailability, therapeutic indices, and antitumor properties, making them ideal for localized delivery of therapeutic genes and drugs to the cancer tumor microenvironment with reduced side effects [199].

**Fig. 17:**
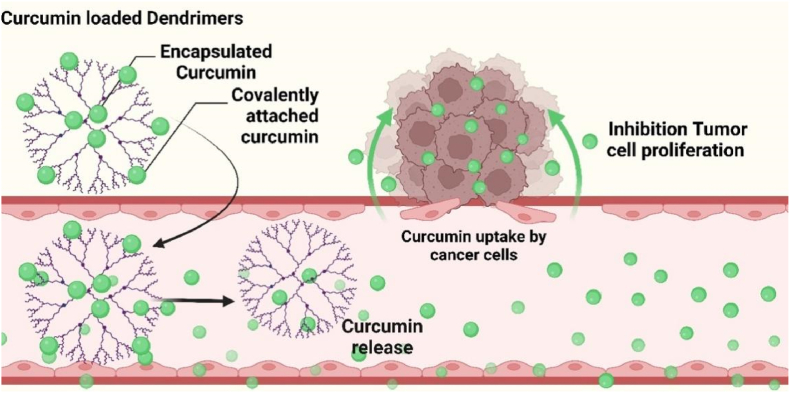
Curcumin-based dendrimers enhanced curcumin uptake and inhibited cancer cell division.

Wei et al. developed amphiphilic dendrimers (AmDM) to create nanomicellar drug delivery systems with high drug-loading capacity (>40 %) for anticancer drugs like doxorubicin (DOX). These AmDM/DOX nanomicelles enhanced drug potency, overcame drug resistance in breast cancer models by increasing cellular uptake and reducing drug efflux, and significantly lowered the toxicity associated with free DOX (Fig. 17) [200].

Dendrimers, particularly as carriers for anticancer drugs, have been widely studied. The delivery of curcumin encapsulated in poly(amino acid) dendrimers demonstrated water solubility and significant cytotoxicity against breast cancer cell lines SKBr3 and BT549 [201]. This suggests that dendrimers-mediated therapy has the advantage of reducing systemic toxicity and causing damage to cancer tissue at the molecular level compared to conventional chemotherapy, resulting in improved patient survival and quality of life [201].

### 2.9 Exosomes

**Fig. 18:**
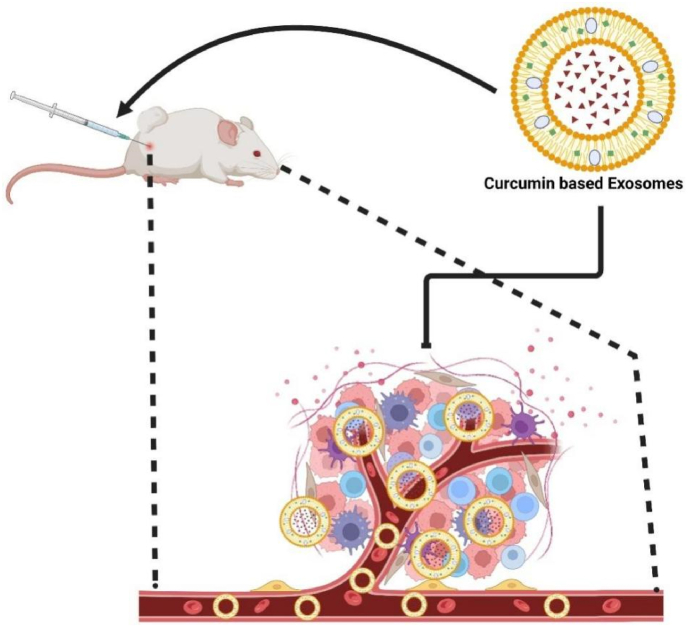
Curcumin-based exosomes enhanced curcumin uptake and inhibited cancer cell division.

Exosomes leverage endogenous trafficking for immune evasion, a unique edge over synthetic carriers. However, low yield and high costs impede clinical adoption. They are most promising for metastatic niches and immunotherapy, where biocompatibility is paramount. Exosomes, secreted by cells with a diameter of about 30–100 nm, carry bioactive molecules such as proteins, lipids, RNA, and DNA. These vesicles play a crucial role in cell communication by transporting materials to recipient cells, influencing their function and behavior [202]. Exosomes have gained significant attention for their role in tumor development, metastasis, and treatment. They can serve as markers for tumor diagnosis and prognosis, as well as therapeutic delivery systems for cancer treatment [203,204]. To enhance curcumin's bioavailability and therapeutic effects, researchers have explored encapsulating curcumin in exosomes. This exosome-based Curcumin delivery system can not only enhance the stability and solubility of Curcumin but also effectively deliver Curcumin to tumor cells through the specific targeting and low immunogenicity of Exosomes, thereby enhancing its anti-tumor activity (Fig. 18) [205,206].

Goyal et al. utilized exosomes as a curcumin delivery system (ExoCUR) for cancer treatment. They found that oral administration of ExoCUR significantly inhibited the proliferation of various cancer cell lines and showed a strong anti-tumor effect in a cervical cancer CaSki tumor xenograft model in vivo. ExoCUR also demonstrated higher anti-inflammatory activity than free curcumin, likely due to the activation of NF-κB in human papillary cancer cells. These findings suggest that exosomes can serve as effective nano-carriers to enhance curcumin's tissue bioavailability and therapeutic efficacy, offering a novel strategy for tumor therapy [205]. Exosomes, as a novel drug delivery system, hold significant potential in enhancing curcumin's efficacy in tumor treatment. By optimizing the preparation process and improving curcumin loading efficiency, safer and more effective exosome-based curcumin delivery systems can be developed, offering more treatment options for tumors.

### 2.10 Nanogels

Nanogels provide stimuli-responsive release and high payloads (30–50 %) but suffer from batch variability. Compared to polymer NPs, they offer better controlled release but require optimization for gelation stability. Their pH-sensitive variants are ideal for melanoma and acidic tumor microenvironments. Nanogels (Ng) are crosslinked polymer-based hydrogel nanoparticles that represent a promising platform for next-generation drug delivery systems, offering advantages such as high drug-loading capacity, low toxicity, and responsiveness to external stimuli. Curcumin-loaded nanogels have shown significant potential in anticancer therapies and drug delivery, enhancing curcumin's bioavailability, targeted delivery, and therapeutic efficacy. These nanogels overcome curcumin's inherent challenges of poor solubility and low bioavailability [207]. Several types of nanogels have been developed for curcumin delivery, including cholesteryl-hyaluronic acid (CHA) nanogels, lipid-polymer-lecithin hybrid nanoparticles, and poly-N-isopropyl acrylamide (p(NIPAm)) nanogels [208].

**Fig. 19:**
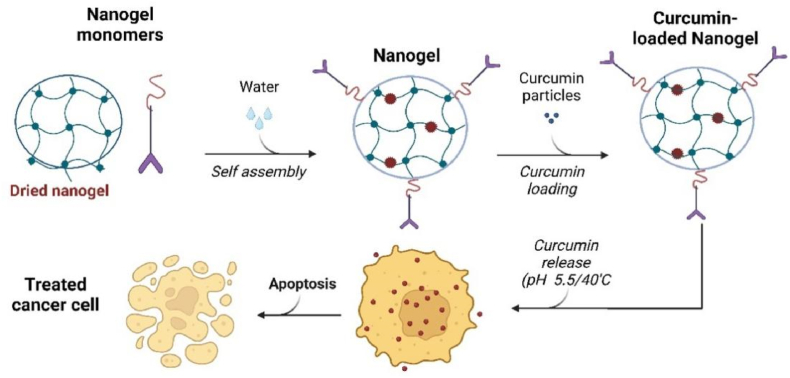
Curcumin-loaded nanogels increased pH and temperature-dependent cellular uptake of curcumin simultaneously and enhanced antitumor activity of tumors by increasing apoptosis of cancer cells.

These nanogel systems demonstrate excellent solubility, sustained drug release, and improved circulation parameters compared to free curcumin [207]. These nanogels also demonstrate improved cellular uptake and targeted delivery to cancer cells, resulting in enhanced cytotoxicity and effective inhibition of tumor growth [209]. Some nanogel systems incorporate dual-responsive properties, such as pH and temperature sensitivity, allowing for controlled release of curcumin under specific conditions (Fig. 19). This feature enables targeted drug delivery to tumor sites, where the acidic microenvironment triggers drug release. Additionally, incorporating gold nanoparticles in some nanogel systems allows for simultaneous drug delivery and photothermal therapy, further enhancing the anticancer efficacy [210].

Mangalathillam et al. developed curcumin chitin nanogel (CCNG) and evaluated its potential for melanoma transdermal therapy. CCNGs demonstrated ideal size, surface properties, excellent drug-loading capacity, controlled release, and effective skin penetration. Confocal microscopy revealed significant internalization of CCNGs in A375 melanoma cells after 6 h, with fluorescence intensity increasing deeper within the cells. In comparison, human dermal fibroblasts exhibited lower internalization levels. Fluorescence imaging of skin samples confirmed CCNGs' deep skin penetration. CCNGs were selectively toxic to melanoma cells without causing hemolysis or toxicity to normal cells. This makes CCNGs promising for transdermal curcumin delivery in treating melanoma and other skin cancers through targeted uptake [211].

Peng et al. developed NG-C nanocapsules for enhanced anti-tumor effects by encapsulating trizimine via microemulsion photopolymerization. To assess in vivo distribution, free Cy5 and NG-Cy5 nanogels (1.5 mg/kg Cy5 dose) were injected into HepG2 tumor-bearing mice. The nanocapsules demonstrated excellent stability and low polydispersity, effectively inhibiting tumor growth, necrosis, apoptosis in HepG2 and HeLa cancer cells, and reducing tumor proliferation [212].

Madhusudana Rao et al. developed interpenetrating polymer network nanogels (IPN-NG) using gelatin and acrylamide-ethylene glycolic acid (AGA) polymers to encapsulate hydrophobic curcumin (CUR) [213]. Tran et al. created pH-sensitive nanocarriers for targeted drug release in the acidic tumor microenvironment. pH-responsive polymers enable rapid drug release in the weakly acidic conditions of tumor tissues while maintaining stability under normal physiological conditions. Polymers like collagen and chitosan are also effective carriers due to their biocompatibility and enhanced delivery efficiency through chemical modifications. Embedding curcumin in collagen nanogels grafted with polyethylene glycol methyl ether (Gel-mPEG) significantly improves its stability and anticancer activity [214]. This nanogel offers optimal size, stability, and controlled curcumin release, boosting its cancer therapy potential. Phan & Santhamoorthy enhanced drug delivery by creating dual-responsive nanocarriers that release drugs rapidly under specific pH and temperature conditions, improving tumor targeting [215]. By utilizing advanced nanotechnologies, such as ph-sensitive collagen-based interpenetrating polymer network nano gels, curcumin's bioavailability and anticancer effects can be significantly improved. These studies provide new ideas for the clinical application of curcumin and open up new directions for developing future anticancer drug delivery systems.

## 3 Bioinspired nanomaterials for curcumin delivery in cancer treatment

**Fig. 20:**
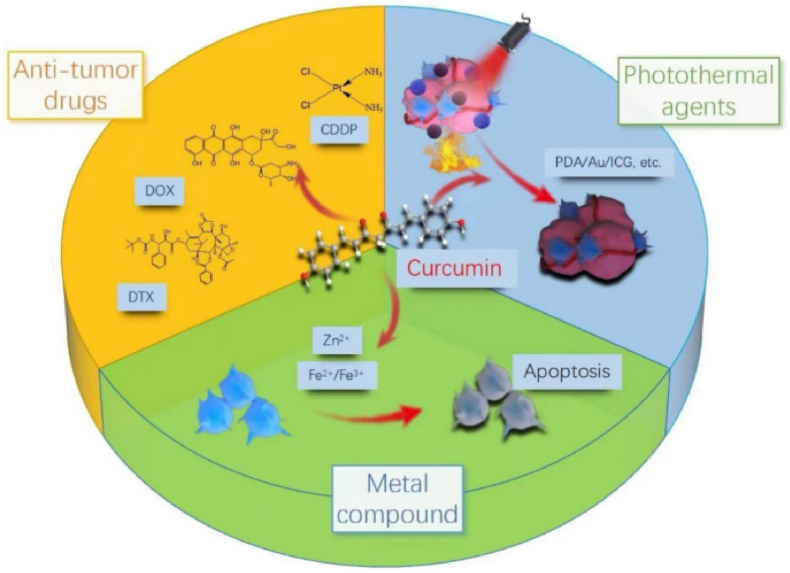
Bioinspired nanomaterial loaded with curcumin for cancer treatment [216].

The limited bioavailability and rapid metabolism of curcumin have driven the development of advanced bioinspired nanodelivery systems [216]. These platforms mimic natural structures or leverage biological components to enhance targeting, stability, and therapeutic efficacy [217,218] (Fig. 20).

### 3.1 Plant-derived nanovehicles

Zhang et al. developed ginger-derived nanovesicles (GDNVs) for curcumin delivery, demonstrating their superiority over synthetic liposomes. These exosome-mimetic vesicles, sized between 100 and 200 nm, achieved a 45 % drug-loading efficiency and exhibited pH-responsive release, with 85 % of curcumin released at the acidic tumor pH (5.5) compared to only 20 % at physiological pH (7.4). In vitro studies revealed a fivefold increase in cellular uptake in HT-29 colon cancer cells due to CD98^−^and CD44-mediated endocytosis. In vivo, GDNVs significantly reduced colitis-associated tumor multiplicity by 55 % in mice, highlighting their potential for targeted therapy [219].

Another innovative approach utilized chlorophyll-inspired porphyrin-phospholipid nanocarriers, as reported by Luo et al. These 150 nm nanoparticles co-loaded curcumin with chlorin e6 for combined chemotherapy and photodynamic therapy. Upon 660 nm laser irradiation, the system generated 90 % reactive oxygen species (ROS), compared to 40 % with free drugs, and exhibited strong synergistic cytotoxicity (combination index = 0.2). In B16F10 melanoma-bearing mice, the formulation reduced tumor volume by 78 %, outperforming curcumin monotherapy (35 % reduction). The system also allowed dual-modal fluorescence and photoacoustic imaging, enabling real-time tracking [220].

### 3.2 Protein-based nanosystems

Safavi et al. engineered human serum albumin (HSA) nanoparticles for colorectal cancer therapy. These 150 nm particles achieved an 8.2 % drug-loading capacity and demonstrated a 12-fold increase in tumor accumulation in CT26 xenografts compared to free curcumin [218]. Additionally, the nanoparticles reversed oxaliplatin resistance by inhibiting P-glycoprotein (P-gp), reducing the IC50 from 48 μM to 9 μM. In survival studies, 80 % of treated mice survived beyond 60 days, compared to only 30 % in the free curcumin group, underscoring the clinical potential of albumin-based delivery [221].

He et al. utilized ferritin nanocages to exploit the acidic tumor microenvironment (TME). These pH-sensitive carriers selectively released curcumin in low-pH conditions, inhibiting STAT3 phosphorylation by 90 % in triple-negative breast cancer (TNBC) models. The ferritin system improved tumor penetration and retention, leading to a 60 % reduction in tumor growth compared to untargeted nanoparticles [222].

### 3.3 Cell-membrane-coated nanoparticles

Chen et al. developed leukocyte-mimetic nanoparticles by coating curcumin-loaded cores with macrophage membranes. These nanoparticles evaded immune clearance and targeted inflamed tumor vasculature, reducing the IC50 from 25 μM (free curcumin) to 8 μM in pancreatic cancer models. The biomimetic design also suppressed metastasis by inhibiting NF-κB and MMP-9 [223].

Fan et al. employed cancer cell membrane camouflage to enhance homotypic targeting. These nanoparticles, derived from 4T1 breast cancer cells, improved tumor-specific uptake and enabled co-delivery of curcumin and doxorubicin. The combination therapy yielded a synergistic apoptosis effect (combination index = 0.3) and suppressed lung metastasis by 70 % in murine models [224].

### 3.4 Microorganism-inspired nanocarriers

Zong et al. (2022) engineered bacterial outer membrane vesicles (OMVs) from *E. coli* to deliver curcumin while activating antitumor immunity. The OMVs selectively targeted TLR4-overexpressing tumors and stimulated a 400 % increase in IFN-γ production, enhancing T-cell responses. In melanoma models, the formulation reduced tumor burden by 65 % and prolonged survival [225].

Liyana EP et al. developed plant-derived virus-like particles (VLPs) from cowpea chlorotic mottle virus for lymphatic delivery. These VLPs improved curcumin bioavailability 20-fold in metastatic lymph nodes and inhibited tumor cell seeding in preclinical models [226].

## 4 Emerging interdisciplinary strategies: Curcumin Nanocarriers Combined with immunotherapy

The integration of curcumin-loaded nanocarriers with immunotherapeutic modalities, such as PD-1/PD-L1 checkpoint inhibitors and CAR-T cell therapy, represents a promising avenue for enhancing antitumor efficacy [227,228]. Recent studies have explored various strategies to harness the immunomodulatory properties of curcumin in combination with these therapies [229].

### 4.1 Combination with PD-1/PD-L1 checkpoint inhibitors

**Fig. 21:**
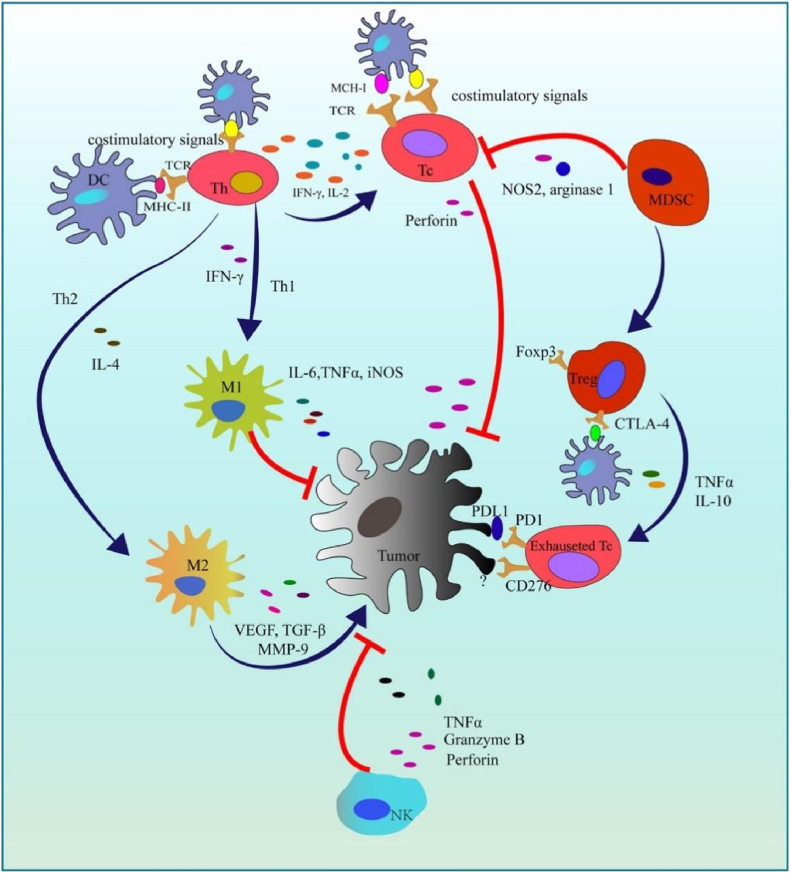
Curcumin Nanocarriers Combined with Immunotherapy inhibited osteosarcoma [231].

Ge et al. developed a pH-sensitive nanocarrier co-delivering curcumin and the PD-L1 inhibitor BMS1166. This system induced immunogenic cell death and enhanced T-cell infiltration in osteosarcoma models, leading to improved antitumor immunity (Fig. 21) [227]. Curcumin-loaded disulfide-crosslinked micelles demonstrated synergistic anticancer efficacy when combined with anti-PD-1 antibodies in a colon cancer model. The micelles improved curcumin's bioavailability and stability, enhancing its therapeutic potential [229]. Highly absorptive forms of curcumin were shown to augment anti-PD-1 therapy by inhibiting STAT3 activation in dendritic cells and tumor cells, thereby restoring T-cell stimulatory activity and enhancing antitumor responses [228]. Intelligent delivery systems targeting the PD-1/PD-L1 pathway have been developed to improve tumor targeting and controllable therapeutic agent release, thereby enhancing the efficacy of immunotherapy [230].

### 4.2 Enhancement of CAR-T cell therapy

PEG-PLGA nanoparticles containing the GSK3 inhibitor SB415286 were found to decrease PD-1 expression and promote the proliferation and survival of CAR-T cells, suggesting a potential role for curcumin-loaded nanocarriers in enhancing CAR-T cell therapy [232]. Nanoparticles have been utilized to enhance cancer immunotherapy in solid tumors by improving drug delivery, modulating the tumor microenvironment, and enhancing T-cell infiltration, which could be beneficial when combined with CAR-T cell therapy [233].

### 4.3 Functionalization strategies for targeted delivery

Folate-decorated PEGylated curcumin-loaded niosomes exhibited enhanced cellular uptake and cytotoxicity in breast cancer cells, indicating the potential for targeted delivery in immunotherapeutic applications [234]. Curcumin–cyclodextrin complexes have been employed in lung cancer models to facilitate targeted drug delivery, leading to significant tumor reduction, highlighting the versatility of curcumin-loaded nanocarriers in various cancer types [232]. The integration of curcumin-loaded nanocarriers with immunotherapeutic strategies, including PD-1/PD-L1 inhibitors and CAR-T cell therapy, offers a multifaceted approach to cancer treatment. These combinations leverage curcumin's immunomodulatory properties and the targeted delivery capabilities of nanocarriers to enhance antitumor efficacy [235]. Continued research into the optimization of these combinatorial strategies is warranted to fully realize their therapeutic potential.

## 5 Positioning curcumin nano-encapsulation within the broader nanomedicine framework

Natural products have emerged as promising candidates in drug discovery owing to their diverse pharmacological properties. However, poor aqueous solubility, low bioavailability, and rapid metabolic degradation limit their clinical application. Nano-encapsulation offers a viable strategy to overcome these limitations [236]. While curcumin has been extensively studied in this regard, several other phytochemicals such as resveratrol, quercetin, berberine, and epigallocatechin gallate (EGCG) have also been investigated using various nanocarrier platforms [237].

Resveratrol-loaded polymeric nanoparticles and lipid-based vesicles have demonstrated enhanced plasma half-life and improved antitumor efficacy. Similarly, quercetin nanoformulations have shown promising results in anti-inflammatory and anticancer models [238]. Berberine, a plant alkaloid with poor oral bioavailability, has been successfully delivered using solid lipid nanoparticles and nanoemulsions. EGCG encapsulated in polymeric micelles and liposomes has shown improved pharmacokinetics and therapeutic outcomes [164,239].

These examples underscore the versatility of nanocarrier-based delivery systems for hydrophobic phytochemicals. The design principles, encapsulation challenges, and biological outcomes observed with curcumin nanocarriers can be extrapolated to these compounds, offering a generalized framework for natural product-based nanomedicine development [240]. Integrating these comparative insights strengthens the translational significance of nanoformulation strategies across a wide range of bioactive molecules [241]. A comparative summary of nanocarrier systems developed for curcumin and other phytochemicals is presented in Table 6.

**Table 6:** Comparative summary of nano-encapsulation strategies for curcumin and other phytochemicals.

Phytochemical	Major Challenges	Nanocarrier Types	Therapeutic Applications	Key Outcomes	Ref
Curcumin	Poor solubility, low bioavailability, rapid metabolism	Liposomes, Polymeric NPs, Solid Lipid NPs, Micelles, Dendrimers	Anti-cancer, Anti-inflammatory, Antioxidant	Enhanced stability, improved pharmacokinetics, targeted delivery	[241]
Resveratrol	Poor solubility, chemical instability, rapid metabolism	Polymeric NPs, Liposomes, Nanoemulsions, SLNs	Anti-cancer, Cardioprotective, Neuroprotective	Prolonged plasma half-life, improved oral bioavailability	[242]
Quercetin	Low water solubility, poor oral absorption	Polymeric NPs, Micelles, Liposomes, Solid Lipid NPs	Anti-inflammatory, Antiviral, Anticancer	Improved solubility, sustained release, enhanced bioavailability	[243]
Berberine	Low bioavailability, poor membrane permeability	SLNs, Polymeric NPs, Nanostructured Lipid Carriers	Anti-cancer, Anti-diabetic, Antimicrobial	Enhanced absorption, prolonged systemic exposure	[244]
EGCG	Instability in physiological conditions, rapid degradation	Polymeric Micelles, Liposomes, Niosomes	Anti-cancer, Cardioprotective, Antioxidant	Improved stability, enhanced therapeutic efficacy	[245]

## 6 Translational progress and clinical potential of curcumin nanomedicine: from preclinical promise to therapeutic applications

### 6.1 Translational progress and clinical evaluation of curcumin nanomedicine

Despite significant preclinical evidence demonstrating the anticancer potential of curcumin-loaded nanomaterials, their translation into clinical application remains limited [76]. Curcumin's poor aqueous solubility, rapid systemic metabolism, and low bioavailability necessitated the development of nano-encapsulation strategies to improve its pharmacokinetics and therapeutic efficacy [246]. Several nanoformulated curcumin products have progressed to clinical trials, reflecting growing translational interest in overcoming these limitations (Table 7).

**Table 7:** Summary of selected clinical trials evaluating nanoformulated curcumin systems.

Formulation	Nanocarrier Type	Clinical Trial ID	Indication	Phase	Status/Outcome
NanoCurc™	Liposomal	NCT01490996	Pancreatic Cancer	II	Safe, increased plasma levels
Lipocurc™	Liposomal	NCT01045318	Healthy Volunteers	I	Well tolerated
CUC-EL	Polymeric Micelles	NCT02163768	Solid Tumors	II	Enhanced half-life

Among the most notable formulations, NanoCurc™, a liposomal curcumin preparation, exhibited improved pharmacokinetics and safety profiles in a Phase I clinical trial for patients with advanced pancreatic cancer (NCT01490996). This study demonstrated that intravenous administration achieved substantially higher plasma curcumin concentrations than oral delivery, with minimal toxicity and preliminary evidence of disease stabilization in some patients [247]. Similarly, Lipocurc™, another liposomal curcumin formulation, was evaluated in a Phase II dose-escalation study in healthy volunteers (NCT01045318), confirming its tolerability and favorable pharmacokinetics, achieving plasma concentrations unattainable via oral curcumin administration [248].

Polymeric nanoparticles encapsulating curcumin have also entered clinical testing. A Phase I study assessing CUC-EL, a curcumin-loaded polymeric micelle system, in patients with advanced solid tumors showed enhanced bioavailability and systemic circulation time compared to free curcumin [3]. While robust antitumor activity was not conclusively established, these studies collectively support the feasibility and safety of curcumin nanoformulations in humans.

Despite these advances, clinical trials evaluating curcumin nanomedicine remain sparse, predominantly confined to early-phase studies with small sample sizes. According to the clinicaltrials.gov database (accessed April 2025), only a limited number of ongoing or completed studies involve nanocarrier-based curcumin systems. Major translational obstacles include challenges in large-scale GMP manufacturing, long-term safety profiling, regulatory hurdles, and consistent batch-to-batch reproducibility [249].

Nevertheless, the development of hybrid nanomaterials integrating therapeutic and diagnostic capabilities (theranostics) is an emerging frontier. Preclinical studies have demonstrated that lipid-polymer and inorganic-organic hybrid nanocarriers can enhance tumor-specific delivery, improve therapeutic outcomes, and enable image-guided interventions. Although these systems show significant promise, their clinical translation has yet to be realized [250].

While curcumin-based nanoformulations display substantial potential in preclinical cancer models, their clinical application remains in an early developmental stage [251]. Future efforts should prioritize multi-center, randomized controlled trials and comprehensive long-term safety evaluations to establish clinical efficacy and translational viability.

### 6.2 Addressing unmet clinical needs through curcumin-based nanomedicine

While curcumin has shown extensive therapeutic potential in preclinical models of cancer, inflammatory, and neurodegenerative diseases, its clinical application remains severely limited due to several pharmacological and therapeutic shortcomings. These include extremely poor aqueous solubility (∼11 ng/mL), low oral bioavailability (<1 %), rapid systemic clearance, poor tissue penetration, and lack of target specificity [247]. As a result, the therapeutic plasma concentrations achieved in human clinical trials have often been insufficient to elicit consistent pharmacodynamic effects, resulting in limited clinical benefit [252].

Curcumin-based nanomedicine platforms have been specifically developed to address these unmet clinical needs. Nanocarriers such as polymeric nanoparticles, liposomes, solid lipid nanoparticles, and dendrimers enhance systemic bioavailability, protect curcumin from metabolic degradation, and improve tissue-specific delivery. For example, liposomal curcumin (Lipocurc™) has demonstrated improved pharmacokinetic profiles in Phase I clinical trials, achieving plasma concentrations >100-fold higher than free curcumin with favorable safety profiles [253].

Additionally, nanomedicine-based systems enable controlled and targeted drug release, reducing off-target effects and toxicity. This is particularly critical in oncology, where EPR-based tumor targeting by nanocarriers has shown to improve intratumoral drug accumulation by 5–10-fold compared to conventional formulations [251]. Moreover, these systems can be engineered for co-delivery of curcumin with chemotherapeutic agents, addressing issues of multidrug resistance and suboptimal monotherapy outcomes.

## 7 Translational barriers, unresolved challenges, and future perspectives in curcumin nanomedicine

Despite substantial preclinical advances, the clinical translation of curcumin-based nanomedicine remains limited due to several persistent challenges. A foremost issue is the scalability and reproducibility of nanocarrier fabrication. While laboratory-scale synthesis often yields promising results, maintaining consistent physicochemical properties, encapsulation efficiency, and batch homogeneity during industrial-scale, GMP-compliant production is difficult. This limitation undermines formulation stability, therapeutic reliability, and commercial feasibility.

Physicochemical stability and long-term storage of nanocarriers present additional hurdles. Many lipid-based, polymeric, and hybrid systems are prone to aggregation, premature drug release, or degradation under physiological conditions, compromising their therapeutic efficacy and shelf life. Furthermore, batch-to-batch reproducibility during scale-up remains a critical challenge for clinical-grade production.

Another unresolved concern is the toxicity and biocompatibility of nanomaterials. Although generally considered safe, certain nanocarriers can elicit immunogenicity, hemolytic effects, and inflammatory responses, especially upon long-term or repeated administration. Moreover, unpredictable biodistribution profiles frequently lead to substantial off-target accumulation, notably in the liver, spleen, and components of the mononuclear phagocyte system, limiting effective tumor-site delivery.

From a regulatory perspective, standardized frameworks specific to nanomedicine, particularly plant-derived formulations like curcumin, remain inadequate. The lack of harmonized guidelines for nanocarrier characterization, safety evaluation, pharmacokinetic assessment, and toxicity profiling creates ambiguity in clinical approval pathways. Extensive toxicological studies, including immunogenicity, genotoxicity, and chronic exposure evaluations, are urgently needed to ensure biosafety.

Additionally, knowledge gaps in understanding the in vivo fate of curcumin-loaded nanocarriers persist. Limited pharmacokinetic studies, predominantly restricted to rodent models, fail to capture human-relevant biodistribution, metabolism, and clearance profiles. Furthermore, most current systems rely on passive or ligand-mediated targeting, whereas stimuli-responsive, environment-adaptive nanoplatforms capable of responding to tumor-specific cues (e.g., pH, enzymes, oxidative stress) are underexplored.

Another limitation is the absence of real-time, non-invasive imaging tools for tracking nanomedicine biodistribution, drug release, and therapeutic response. Integration of multimodal imaging systems (PET/CT, MRI-nanoparticle hybrids) would facilitate dynamic monitoring of nanocarrier behavior in vivo.

### 7.1 Future perspectives

To overcome these challenges, future research should focus on.

• Establishing standardized, scalable, and cost-effective manufacturing protocols ensuring physicochemical stability and batch homogeneity.
• Conducting comprehensive biodistribution, toxicokinetic, and long-term biosafety studies in human-relevant models.
• Designing multifunctional, stimuli-sensitive, and theranostic nanocarriers for tumor-specific, image-guided curcumin delivery.
• Harmonizing regulatory guidelines and evaluation frameworks for nanomedicine approval.
• Integrating advanced imaging modalities for real-time, quantitative monitoring of nanomedicine fate and therapeutic response.
• Exploring personalized and precision medicine approaches by tailoring nanocarriers to patient-specific tumor profiles and drug resistance mechanisms.
• Developing combination strategies integrating curcumin nanomedicine with immunotherapy and gene therapy, leveraging curcumin's immunomodulatory and tumor-suppressive properties.

Addressing these translational barriers through multidisciplinary, collaborative efforts will be essential to bridge the gap between preclinical promise and clinical application, ultimately realizing the therapeutic potential of curcumin-based nanomedicine in oncology and beyond.

## 8 Conclusion

Nanomaterial-encapsulated curcumin has significantly enhanced its bioavailability, stability, and targeted delivery for cancer therapy. Various nanocarriers amplify curcumin's therapeutic potential, offering promising results in preclinical studies. This approach holds great potential for personalized cancer treatment by enabling customized delivery systems based on tumor characteristics and patient needs. Furthermore, integrating curcumin with other therapies could offer synergistic effects, improving overall treatment outcomes. However, further research and clinical validation are needed to address challenges such as optimizing delivery systems, ensuring safety, and confirming efficacy in clinical applications. Moreover, the principles derived from curcumin nanoformulation studies, including carrier selection, surface modification, and bioavailability enhancement strategies, offer valuable guidance for the design of nanocarrier systems for other hydrophobic natural products such as resveratrol, quercetin, berberine, and EGCG. This integrative approach reinforces the broader applicability of nanomedicine-based phytochemical delivery strategies in modern therapeutic interventions.

## CRediT authorship contribution statement

**Yuxing Yan:** Writing – original draft, Software, Data curation, Conceptualization. **Kulsoom:** Writing – original draft, Software, Formal analysis. **Yanbo Sun:** Software, Data curation. **Yingjie Li:** Software, Formal analysis. **Zhenlong Wang:** Supervision, Project administration. **Li Xue:** Supervision, Project administration. **Fu Wang:** Writing – review & editing, Supervision, Project administration, Funding acquisition.

## Funding

This work was supported by the 10.13039/501100001809National Natural Science Foundation of China (No. 32271512), the Natural Science Basic Research Program of Shaanxi (No. 2022JC-56, 2023-JC-ZD-43).

## Declaration of competing interest

The authors declare that they have no known competing financial interests or personal relationships that could have appeared to influence the work reported in this paper.

## Data Availability

The data are included in this manuscript.
